# Production of Aromatic Compounds by Catalytic Depolymerization of Technical and Downstream Biorefinery Lignins

**DOI:** 10.3390/biom10091338

**Published:** 2020-09-18

**Authors:** Alfonso Cornejo, Fernando Bimbela, Rui Moreira, Karina Hablich, Íñigo García-Yoldi, Maitane Maisterra, António Portugal, Luis M. Gandía, Víctor Martínez-Merino

**Affiliations:** 1 Institute for Advanced Materials and Mathematics (InaMat2) and Department of Sciences, Universidad Pública de Navarra, E31006 Pamplona, Spain; karina.hablich@unavarra.es (K.H.); garciayoldi@gmail.com (Í.G.-Y.); maitane.maisterra@unavarra.es (M.M.); lgandia@unavarra.es (L.M.G.); merino@unavarra.es (V.M.-M.); 2 CIEPQPF, FCTUC, Department of Chemical Engineering, University of Coimbra, Rua Sílvio Lima, Pólo II—Pinhal de Marrocos, 3030-790 Coimbra, Portugal; ruimoreira@eq.uc.pt (R.M.); atp@eq.uc.pt (A.P.)

**Keywords:** lignin, depolymerization, homogeneous and heterogeneous catalysis, bio-based aromatic compounds

## Abstract

Lignocellulosic materials are promising alternatives to non-renewable fossil sources when producing aromatic compounds. Lignins from *Populus salicaceae*. *Pinus radiata* and *Pinus pinaster* from industrial wastes and biorefinery effluents were isolated and characterized. Lignin was depolymerized using homogenous (NaOH) and heterogeneous (Ni-, Cu- or Ni-Cu-hydrotalcites) base catalysis and catalytic hydrogenolysis using Ru/C. When homogeneous base catalyzed depolymerization (BCD) and Ru/C hydrogenolysis were combined on poplar lignin, the aromatics amount was ca. 11 wt.%. Monomer distributions changed depending on the feedstock and the reaction conditions. Aqueous NaOH produced cleavage of the alkyl side chain that was preserved when using modified hydrotalcite catalysts or Ru/C-catalyzed hydrogenolysis in ethanol. Depolymerization using hydrotalcite catalysts in ethanol produced monomers bearing carbonyl groups on the alkyl side chain. The analysis of the reaction mixtures was done by size exclusion chromatography (SEC) and diffusion ordered nuclear magnetic resonance spectroscopy (DOSY NMR). ^31^P NMR and heteronuclear single quantum coherence spectroscopy (HSQC) were also used in this study. The content in poly-(hydroxy)-aromatic ethers in the reaction mixtures decreased upon thermal treatments in ethanol. It was concluded that thermo-solvolysis is key in lignin depolymerization, and that the synergistic effect of Ni and Cu provided monomers with oxidized alkyl side chains.

## 1. Introduction

Lignin is a very complex natural amorphous polymer, mainly composed of three different phenylpropanoid units: p-coumaryl alcohol, sinapyl alcohol and coniferyl alcohol [1,2]. In spite of the relatively simple structure of these three monomeric units, their layout is highly complex, conforming an intricate tridimensional structure of distinct and chemically different motifs and natural alternative monomers as a consequence of their random radical polymerization [3]. These moieties and distinctive bonds are most responsible for the unique structures and properties of lignins rather than the three main phenolic building blocks, and they are often the key to elucidate lignin morphology, physicochemical properties and reactivity.

The structure and properties of native lignin are altered upon lignin isolation, as can be seen in Table 1, which presents different methodologies for lignin isolation. Great amounts of lignin-rich streams are produced, foremost in the pulp and paper industry by Kraft pulping [4]. More recently, large-scale processes for producing biofuels, e.g., second-generation cellulosic bioethanol, have generated large amounts of downstream biorefinery effluents [5,6]. Due to the inherent recalcitrance of lignins, their main industrial use is as low-grade fuel to produce energy [7], whilst allowing for the recovery of Kraft cooking reagents [8]. However, the large amounts of lignin produced in biorefineries generate surplus stocks that often exceed the energy demands of those plants [5]. The structure and properties of both Kraft and downstream biorefinery lignins are very different to those of the native lignins.

Other alternatives for obtaining lignins include different organosolv (Org) methods which use organic solvents, either pure or in aqueous solutions [9,10], sometimes combined with other reactants, as in the soda-organosolv method (Seol) [11]. Due to the mild conditions used, the structure of organosolv lignins is very similar to their native structure. These isolation methods yield lignins often referred to as technical lignins.

Given the chemical richness derived from its highly complex structure, lignin depolymerization is seen as an alternative to petrochemical industry for the production of aromatic compounds. Aromatics including monomeric phenols and other related aromatic compounds have been extensively sought after by different studies on catalytic depolymerization of lignin [15,16], some of which date back to 1963 [21]. Much effort has been made to develop routes for valorizing lignin into higher value-added chemicals and products, as can be deduced from the comprehensive reviews on the topic [2,3,4,7,12,22,23,24,25,26,27,28,29,30,31,32,33,34,35], many of which have been successful and the number of proven commercial applications and products from technical lignin is vast [36].

Most of the studies on catalytic technical lignin depolymerization rely on the heterogeneous catalysis for obtaining bioaromatics and other value-added compounds. In this context, Ru supported on carbon (Ru/C) has become the most widely used catalyst for the hydrogenolysis of technical lignins owing to its superior selectivity for cleaving C_aryl_-O bonds while maintaining a moderate activity for the hydrogenation of aromatic rings in comparison to other catalysts [37]. Ru/C is commonly used in combination with alcohols, mainly methanol and ethanol under supercritical conditions [28]. The main reasons for this is the rapid heat transfer, the high solubility of technical lignins and the reaction mechanism itself: alcohols not only can act as hydrogen donors but also as nucleophiles initiating the C-O-C bond cleavage [38]. Furthermore, ethanol also acts as a capping agent and formaldehyde scavenger through the formation of n-propanol thus preventing repolymerization reactions [39,40]. The combination of Ru/C catalysts and NaOH under methanol solvent medium has proven to be effective in the depolymerization of organosolv pine-derived lignin owing to the synergistic catalytic effect of NaOH and Ru/C [41].

Reductive catalytic fractionation (RCF) using Ru/C catalysts, including catalytic hydrogenolysis in the liquid phase [2,16,17], is an interesting approach to fractionate lignocellulosic residues. To this end, delignification is conducted via thermo-solvolysis readily followed by reductive stabilization of lignin. Using a combination of a Ru/C catalyst, methanol as solvent and pressurizing with hydrogen at 30 bar, delignification of hardwoods, such as birch sawdust, can be successfully attained. In this way, depolymerization of lignin fragments effectively leads to high yields of methoxyphenolic monomers, dimers and oligomers [16]. However, recent studies have revealed that, in the presence of bases such as KOH, RCF of birch wood suffers from partial repolymerization of lignin. This lowers the yield of phenolic monomers, but the selectivity to desirable C2-fragmented phenols can be favored over that to C3 phenols [17]. Therefore, adding bases to the reaction medium may hamper lignin depolymerization but enhances selectivity toward the desired products; hence, a trade-off has to be achieved by an appropriate selection of reactions conditions and process design.

Amidst the different proposed routes, biorefinery strategies that comprise catalytic depolymerization of lignin of downstream biorefinery lignins are of great interest, because they allow producing different organic compounds that can have potential applications and industrial use. However, the high recalcitrance of these lignins restricts most of the research to homogeneous BCD [42], whereas heterogeneous catalysis has been considerably much less investigated. Ru/C was selected, among other Ru-based catalysts, for conducting the reductive depolymerization of an industrial lignin-containing stillage derived from lignocellulosic bioethanol production [5]. Ru/C yielded the highest amount of monomeric phenols, but the yields were somewhat low, probably due to repolymerization reactions. In a previous work [6], samples from lignin-rich biorefinery streams from poplar and pine woodchips were successfully depolymerized under BCD conditions, obtaining remarkable monomer yields by doing a careful selection of operating conditions.

Heterogeneous BCD using double-layered hydroxides known as hydrotalcites (HTC) has emerged as a promising alternative in recent years. Ni-functionalized HTCs have proven to be effective in cleaving C-O bonds of model compounds and in lignin depolymerization [43]. Up to 7–9 wt.% monomer yield was obtained due to the effect of nitrates intercalated as counter ions between the brucite-like layers that constitute the HTCs structure [44]. The efficiency of Ni-modified HTC catalysts is attributed both to their capability of specifically hydrolyzing the carbon-hydroxyl linkage at the side chain into alkanes and to the presence of strong binding sites for ether linkages, thus achieving their specific cleavage [45]. HTCs containing copper (Cu-HTCs) have also proven to be effective in lignin depolymerization under supercritical ethanol (scEtOH) at 573 K [46] and 613 K [47], giving monomer yields around 20 wt.% and 30 wt.%, respectively. However, these fragments are further alkylated or hydrodeoxygenated by the solvent [39], which effectively reduces the aromatic monomers yield. Noteworthy, HTC-Cu catalysts under scEtOH at 653 K produced up to 86 wt.% alkylated monomers in lignin depolymerization, with high degree of hydro-deoxygenation and hydrogenation of the aromatic ring [39]. It has been also reported that HTCs-Cu promote lignin depolymerization in supercritical methanol (scMeOH), preventing char formation [48].

Bimetallic catalysts combining Ni with noble metals and transition metals like Fe, Mo and Ti have been proposed in different studies to explore synergistic effects, thus seeking to increase the reactivity and selectivity [31]. Zhai et al. found that bimetallic NiFe supported on active carbons could effectively cleave ether linkages whilst suppressing hydrogenation of aromatic rings [49]. It was proposed that the formation of NiFe alloys tuned the reactivity of the active sites. HTC-Ni catalysts have already been used in the cleavage of model lignin dimeric compounds as well as in lignin-rich biorefinery streams derived from corn stover [44]. More specifically, enzymatically hydrolyzed lignins, some of which had been pretreated with diluted acids, were subjected to depolymerization, finding that the catalyst was effective in cleaving ether linkages, as evidenced by the formation of substantial amounts of 4-vinylphenol (4-VP) produced by the decarboxylation of p-coumaric acid. To the best of our knowledge, bimetallic HTC-NiCu catalysts have not been used in the depolymerization of lignins.

In this contribution, homogeneous and heterogeneous catalytic methods were developed to depolymerize downstream biorefinery and technical lignins. The resultant reaction mixtures were thoroughly characterized to accurately determine the aromatic monomer yield and selectivity, the poly-(hydroxy)-aromatic fractions apparent masses and the extent of side-reactions.

## 2. Materials and Methods

All high-purity liquid and solid chemicals were purchased from Carlo Erba, Fisher Scientific and Sigma-Aldrich and used as received. High-purity gases were supplied by Nippon Gases Spain. Synthetic hydrotalcite (Mg_6_Al_2_(CO_3_)(OH)_16_·4H_2_O), used as support for preparing the catalysts, was purchased from Sigma Aldrich (P.N. 652288). A commercial 5% Ru/C (50% water wet) catalyst was purchased from Alfa Aesar (P.N. 044338.14).

Poplar (*Populus* sp.) was an industrial residue kindly provided as sawdust by Garnica Plywood Inc. (Baños de Rio Tobia, Spain). Poplar sawdust was air dried at room temperature (~295 K) for 72 h, then it was sieved to obtain a particle size distribution comprised between 0.5 and 1.0 mm (35–18 mesh). After sieving, the air-dried material was further dried in an oven at 376 K for 24 h. Biorefinery downstream lignins from pine (*P. radiata*) and poplar (*Populus* sp.), named as BioA and BioB, respectively, were kindly provided by CENER’s Biorefinery and Bioenergy Centre (Bio2C, Aoiz, Spain). Both *P. radiata* and *Populus* sp. were used as feedstocks in the production of 2 G bioethanol in a biorefinery, obtaining BioA and B as byproducts. The whole process for obtaining BioA and B starting solids is detailed in a previous study [50], although succinctly described below (please see Section 2.1). BioA and B lignins were received in powder form and used without further treatments. *P. pinaster* wood (PPW) chips were kindly supplied by Europac (Deocriste, Portugal). Prior to fractionation, as received PPW chips were air-dried and ground in a Retsch Cutting Mill SM 100 with a sieve of square holes of 4 mm. Further details can be found elsewhere [51].

### 2.1. Lignin Isolation and Characterization

**Scheme 1:**
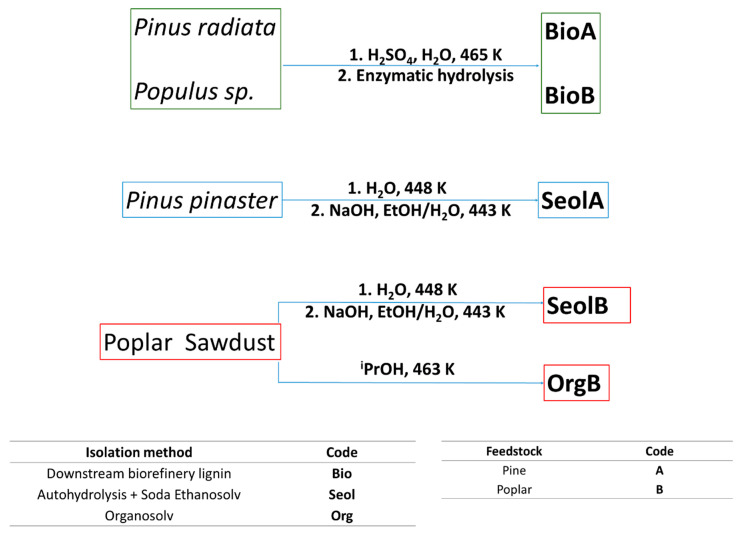
Strategies for the isolation of lignins from the feedstocks selected in this work.

Different strategies were used in this study for obtaining lignin from the four feedstocks described above (see Scheme 1), namely autohydrolysis followed by soda ethanosolv (SeolA and B lignins for pine and poplar respectively), direct organosolv (OrgB, for poplar organosolv) using isopropanol, and the use of biorefinery downstream lignins (BioA and B lignins for pine and poplar respectively).

Biorefinery downstream lignins, BioA and B, were obtained after acidic pretreatment at 465 K for 5 min of the pine and poplar feedstocks, respectively, which allowed to separate their corresponding hemicellulosic fractions and to valorize them thereafter. The resulting solids presented high accessibility to the cellulosic fraction that was hydrolyzed to glucose using an enzymatic cocktail, whilst yielding BioA and B as downstream biorefinery lignins [6,50].

Technical lignins, OrgB, SeolA and B, were isolated in the laboratory. SeolA and B were obtained from *P. pinaster* and waste poplar sawdust, respectively. Feedstocks were subjected to autohydrolysis at 448 K during 30 min to remove hemicelluloses, followed by soda ethanosolv digestion at 443 K during 90 min, using a NaOH load of 30 wt.% (on a wood basis) and a biomass to solvent ratio of 1:8 (*w*/*w*). SeolA isolation experiments were performed in a 1 L autoclave reactor (Parr Instruments), agitated by a double six-blade propeller while SeolB isolation experiments were performed in a 150 mL autoclave (Parr instruments) agitated by a four-blade propeller. On the other hand, OrgB was directly obtained following a direct organosolv procedure using several combinations of alcohols and acidic catalysts. Amongst them, organosolv digestion with ethanol and isopropanol at 463 K provided the best lignin yields. However, DOSY (see Figure S8 in Supporting Information) evidenced that OrgB obtained with ethanol presented more impurities, including some cellulosic residues. Therefore, OrgB obtained with isopropanol was selected for conducting the depolymerization assays.

Lignin content was determined according to NREL/TP 510-42618. The ratio of syringyl (S) and guaiacyl (G) units, the S/G ratio, was determined as described in the literature in BioA and B (see Supporting Information SI.1) whereas ^31^P NMR was used in the rest of lignins (see Supporting Information SI.2).

Average molecular weight (M_w_) and number molecular weight (M_n_) were determined using Size Exclusion Chromatography (SEC, see details in Supporting Information SI.2), in lignins and depolymerization products. Additionally, the apparent masses were estimated using Diffusion Ordered Spectroscopy NMR (DOSY, see Supporting Information SI.3 for details). DOSY spectroscopy has been used in this work to monitor depolymerization reactions and to selectively determine the apparent mass of the poly-(hydroxy)-aromatic fraction in the reaction mixtures. Apparent masses were estimated using two calibration curves that correlated the average log D of the diffusion traces and their log MW (see reference [6] for details). The calibration curves were calculated using two different families of standards. The first was polystyrene (PS) standards that corresponded to dispersion forces, and the second was polyethylene glycol (PEG) and dimeric and monomeric phenolic standards—referred to as a PEG calibration curve)—that corresponded to Van der Waals and even hydrogen bond interactions with the solvent. Lignin and depolymerization products have been also analyzed using Heteronuclear Single Quantum Coherence Spectroscopy NMR, HSQC, and Total Correlation HSQC NMR spectroscopy, HSQC-TOCSY (see Supporting Information SI.3 for experimental details).

#### 2.1.1. Organosolv Method (OrgB)

The extraction of lignin from waste poplar sawdust was carried out in a 200 mL high-pressure stainless-steel reactor (Berghoff BR-300) equipped with a magnetic stirrer and a Teflon liner. In a typical isolation reaction, a 10% (*w*/*v*) suspension of poplar sawdust in isopropyl alcohol was pressurized with nitrogen (20 bar), stirred at 463 K for 3 h, and then allowed to cool down to room temperature. The reaction mixture was filtered, and the solid residue was washed thrice with isopropyl alcohol (3 × 20 mL). The filtrate was concentrated to ca. 20 mL in a rotary evaporator, then poured onto distilled water (200 mL) at 273 K under vigorous stirring. The mixture was allowed to settle overnight, and the resulting brown precipitate was collected by filtration and vacuum dried at room temperature overnight, thus yielding OrgB.

#### 2.1.2. Autohydrolysis and Soda Alkaline Ethanosolv Method (SeolA and B)

SeolA and B lignins were obtained by fractionation of *P. pinaster* wood and poplar sawdust, respectively. Aqueous suspensions of the feedstocks in Milli-Q H_2_O (10% *w*/*v*) were prepared and readily put into the reactor. The suspensions were stirred at 448 K for 30 min and then allowed to cool down to room temperature. The reaction mixture was then filtered and the solid was washed with H_2_O.

The solid was re-suspended in a 35% ethanol-water mixture (biomass-to-solvent mass ratio of 1:8) and reacted with 30 wt.% NaOH (on solid basis) at 443 K for 90 min, and then allowed to cool down again. The reaction mixture was then filtered, and the solids were washed with H_2_O. The combined filtrates were concentrated by rotary distillation. The resulting solution was brought to pH 5 upon addition of H_2_SO_4_ and allowed to settle down. The resulting solids were collected by filtration, re-suspended in H_2_O and collected again by filtration. The as-obtained lignin was dried at 313 K for 7 days.

### 2.2. Preparation and Characterization of Hydrotalcite-Supported Metal Catalysts (HTC-M)

A set of five HTC-M solids were prepared by impregnation with a total nominal metal content of 5 wt.%. Varying amounts of Ni and Cu were used in the preparation of the different HTC-M catalysts, resulting in HTC0-5, where the figure corresponds to the nominal nickel loading.

The hydrotalcite-supported metal catalysts were prepared by wet impregnation of a commercial hydrotalcite. The support was calcined at 723 K for 24 h in a muffle furnace, thus obtaining the calcined support (HTC). Then, the HTC was rehydrated with H_2_O for 1.5 h and subjected to ultrasonic vibration for 5 min. Known amounts of stock solutions of Ni(NO_3_)_2_·6H_2_O and Cu(NO_3_)_2_·3H_2_O in ethanol were then added to the rehydrated HTC, to obtain catalysts with different nominal metal loadings: 5 wt.% Ni, (HTC-5), 4 wt.% Ni—1 wt.% Cu, (HTC-4), 2.5 wt.% Ni—2.5 wt.%-Cu (HTC-2.5), 1 wt.% Ni—4 wt.% Cu (HTC-1) and 5 wt.% Cu (HTC-0). The resulting suspensions were then stirred at room temperature for 5 min. The solvent was then evaporated, and the resulting solids were grinded into powder form for obtaining the different metal-HTC catalysts.

The resulting solids were characterized by N_2_ physisorption, Inductively Coupled Plasma-Optical Emission Spectrometry (ICP-OES) and X-ray Diffraction (XRD). N_2_ adsorption-desorption isotherms were determined in a Micromeritics Gemini V 2380 static volumetric analyzer at 77 K. The samples were previously degassed at 473 K for 2 h under a N_2_ gas flow. The specific surface area (m^2^/g) of the samples was determined by the Brunauer-Emmett-Teller (BET) method, whereas the specific pore volume and the average pore size were calculated by the Barrett–Joyner–Halenda (BJH) method. The elemental analysis of the catalysts was done by means of optical emission spectrometry with inductively coupled plasma (ICP-OES, Thermo Elemental IRIS INTREPID RADIAL, equipped with a Timberline IIS automatic). XRD analyses were done using a D-Max 2500 Rigaku diffractometer with CuKα radiation at 40 kV and 80 mA and scanning 2θ from 5 to 95°. Both ICP-OES and XRD analyses were carried out at the “Servicio de Apoyo a la Investigación” of the Universidad de Zaragoza (Zaragoza, Spain).

### 2.3. Lignin Depolymerization

**Scheme 2:**
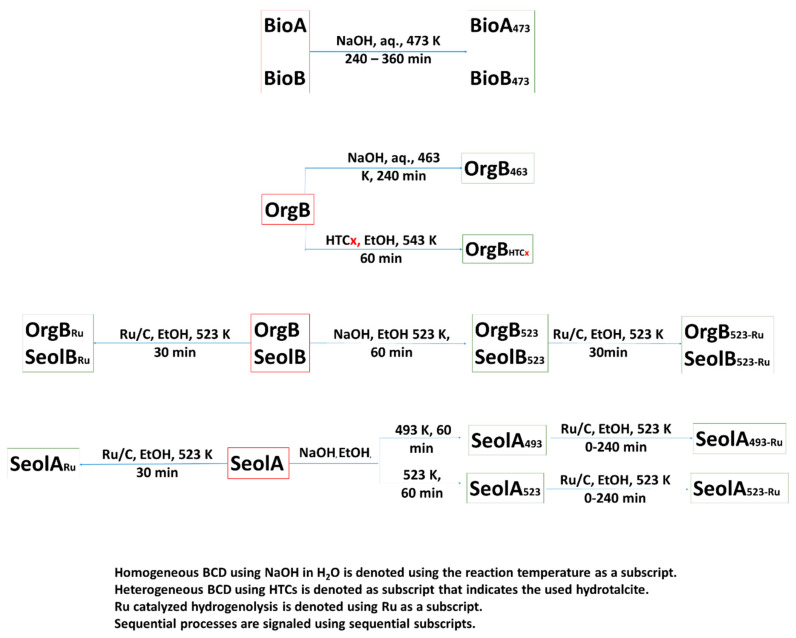
Lignin depolymerization strategies.

Different strategies were planned for each of the isolated lignins depending on their physico-chemical properties (see Scheme 2). These will be described in detail in the following subsections, including homogeneous base catalyzed depolymerization using aqueous NaOH, NaOH in ethanol-water mixtures, and heterogeneously catalyzed depolymerization using Ru/C or Ni-, Cu- and NiCu-HTCs.

#### 2.3.1. Base Catalyzed Depolymerization Using Aqueous NaOH or NaOH aq. (BioA473, BioB473, OrgB463)

Depolymerization reactions were carried out using aqueous solutions of NaOH in a 100 mL batch stirred pressurized stainless-steel vessel reactor (Autoclave Engineers, EZ100RXR) equipped with a PID controller. Following this, 50 mL of lignin suspension in 0.25 M NaOH aq. (1% *w*/*v*; pH 13.4, NaOH/solid ratio 25:1) was pressurized with N_2_ (20 bar) and stirred at 463 K (OrgB) or 473 K (BioA or B) during 240 min. The resulting solutions were filtered, and the liquid was acidified to pH 2 upon addition of 1 M HCl. Then, 25 mL of a solution of bromobenzene (0.0015 M) in ethyl acetate, and additional ethyl acetate when necessary, was then added and the organic phase was separated. 1.0 mL of the organic phase was taken for the quantification of monomeric phenols by GC-FID. The identity of the monomeric phenols was confirmed by GC-MS (see Supporting Information SI.2). The rest was evaporated to dryness and the yield of the oily fraction recovered (herein referred to as bio-oil) was gravimetrically calculated and used for further characterization (Supporting Information SI.2 and SI.3).

#### 2.3.2. Base Catalyzed Depolymerization Using NaOH in Aqueous EtOH or EtOH aq. (OrgB523, SeolA493, SeolA523 and SeolB523)

Lignin (1.900 g) was suspended in a NaOH (1.800 g) solution in EtOH/ H_2_O (35 wt.%, 50 mL)_._ The mixture was pressurized with nitrogen (20 bar), readily stirred at 493 K or 523 K in an Autoclave Engineers reactor for 1 h and then allowed to cool down. The reaction mixture was filtered, and the solids were washed. The ethanol in the combined filtrate was removed by rotary evaporation and the resulting solution was brought to pH 2 upon addition of 1 M HCl. The subsequent procedure for quantifying monomeric phenols in the samples by GC-FID and to determine the bio-oil yield is the same as that detailed in the previous subsection (see above).

#### 2.3.3. Depolymerization Using Ru/C or HTC-M Catalysts

A solution of lignin or solids SeolA_493_ or A_523_ (250 mg) in ethanol (50 mL) and a load of catalyst (250 mg of HTC-M or 500 mg of Ru/C -50% water wet-) were placed in the autoclave reactor, which was then sealed. Air was purged with nitrogen thrice and the reactor was subsequently pressurized with nitrogen (20 bar) or hydrogen (30 bar). The reaction medium was then stirred at 523 K during 30 min (Ru/C catalyst) or 543 K during 60 min (HTC-M catalyst). The reactor was then allowed to cool down, the reaction mixture was filtered and the solid was washed with ethanol (20 mL). The combined filtrates were concentrated by rotary evaporation and then distilled water (50 mL) was added. The next steps for quantifying monomeric phenols in the samples are the same as those detailed in the two previous subsections (see above).

## 3. Results

### 3.1. Characterization of the Different Lignins

Table 2 shows the main characteristics of the lignins used in this study. As expected, the purity of the technical lignins SeolA, and OrgB was much higher (98.0 wt.% and 96.1 wt.%, respectively) than those of downstream biorefinery lignins, BioA and B (51.1 wt.% and 69.1 wt.%, respectively). It is important to note that BioA and B presented a relatively high sulfur content coming from the acidic thermal pretreatment with sulfuric acid [50], which causes some sulfonation in the aromatic rings. Most of the impurities in biorefinery lignins, 38.8 wt.% in BioB and 23.2 wt.% in BioA, corresponded to glycans that were not fully removed upon enzymatic hydrolysis. Opposite to this, neither hemicelluloses nor celluloses or glycans were detected in SeolA and B, whereas only 0.4 wt.% of glycans were found in OrgB. In the case of SeolB, lignin content in the isolated solid was surprisingly low, 68.7 wt.%, with a high ash content, 30.1 wt.%. The reason for this high content in ashes could be a non-efficient removal of salts formed during lignin isolation.

According to the SEC analyses, downstream biorefinery lignins (see Figure S2 in Supporting Information) presented much broader mass distributions than technical lignins. BioB presented its maximum at 1038 Da and BioA at 228 Da. However, fractions beyond 32000 Da were found in both cases. Although SEC for biorefinery lignins was carried out using 0.1% LiBr in *N,N*-dimethylformamide, neither BioA nor B could be completely solubilized. This is probably the reason for the high dispersity index found for these solids. Presumably, low molecular weight fractions are much more soluble and provide the relatively low M_n_ in both cases, 535 Da and 414 Da, respectively. Size distributions in SeolA, B and OrgB were much narrower, as it is pointed by the dispersity index, which is, in all cases, in the range of 1.3 to 1.4. SEC chromatograms for OrgB and SeolB (see Figure S2 in Supporting Information) presented similar profiles. M_n_ values were similar in both cases and no significant fractions could be detected at mass values beyond 4500 Da. SeolA also showed a much narrower distribution than BioA, presenting M_n_ values (2209 Da) which were much higher than those of OrgB and SeolB. Size distribution in SeolA, however, was broader and lignin fragments could be detected at mass values of ca. 8000 Da.

In a recent work [6] we have shown that DOSY spectroscopy can be used to selectively determine the apparent mass of poly-(hydroxy)-aromatic fractions in BCD reaction mixtures. This estimation was carried out after proper calibration of log D *vs* log MW of two different families of standards, polystyrene (PS) and poly-ethylene glycols and monomeric bioaromatics (PEG curve). These two families of standards accounted for different solute-solute and solute-solvent interactions. DOSY was particularly useful in the characterization of lignins. The Stejskal-Tanner equation (Supporting Information SI.3) permitted estimating the diffusion coefficients (D) of the diffusion traces that appear in the aromatic, methoxy and aliphatic regions of the spectra and, through a proper calibration, correlating D to the apparent masses. This is particularly interesting in BioA and B lignins, whose SEC analyses were not reliable due to their low solubility in the eluent. The solubility of BioA and B was very low also in deuterated dimethylsulfoxide (DMSO-*d_6_*) even after acetylation (see Supporting Information for details), which complicated both the HSQC (see Figure S13 in Supporting Information) and the corresponding DOSY analyses (see Figure S3 in Supporting Information). Assuming that only lignin presents aromatic moieties in the analyzed samples, D for the aromatic region can be associated with the apparent masses of lignin. Apparent masses in the aromatic region for BioA were 1274 Da using PS calibration curves. In the case of acetylated BioB, apparent masses were graphically determined because experimental and theoretical area intensities determined by the Stejskal-Tanner equation did not present a good correlation (see Table S2 in Supporting Information). Apparent masses in the aromatic region were 1630 Da, but cellulosic residues presented ^1^H signals in the range of 4.0–5.0 and 3.0–3.8 ppm, with higher intensity than those from lignin due to their higher solubility. Apparent masses for the saccharide region (4.5–3.0 ppm) ranged from 317 Da to 1796 Da in BioB, which is concordant with the SEC measurements (see Table 2 and Figure S2 in Supporting Information). Intense signals around 2.0 ppm appeared due to acetylation of lignin and the saccharide residues. Aliphatic traces at high fields (1.0 ppm–2.0 ppm) corresponded, almost exclusively to alkyl chains and solvent residues from acetylation (see Figure S3 in Supporting Information).

Poplar lignins isolated via organosolv, OrgB, and pine and poplar lignins isolated by autohydrolysis followed by soda ethanosolv, SeolA and B respectively, were much more soluble in DMSO-*d_6_* than biorefinery lignins. Average diffusion coefficients were determined using the Stejskal-Tanner equation for the aromatic region of OrgB and SeolB that corresponded with apparent masses of 1445 Da and 1477 Da, respectively, which are in the range of M_n_ determined by SEC (1230 Da and 1295 Da respectively). Correlation in the Stejskal-Tanner equation was not optimal for SeolA and the diffusion coefficient was estimated graphically from the DOSY spectrum processed using TOPSPIN 3.6.2 corresponding to an apparent mass of 2122 Da. Most of the diffusion traces in DOSY spectra for technical lignins (see Figure S4 in Supporting Information) turned up in the aromatic region and were thus assigned to the aromatic hydrogen atoms of lignins. Therefore, average apparent masses for lignins estimated by DOSY presented only slight differences with M_n_ determined by SEC. OrgB presented some traces in the 4.0 ppm–5.0 ppm range, which can be associated to hemicelluloses and/or celluloses, as it is shown in its compositional analysis (Table 2), centered in in the range of 1000–1340 Da. Nevertheless, the aliphatic region presented hydrogen signals in the range of 540–914 Da according to PS calibration (see Figure S4 in Supporting Information). These hydrogen signals may correspond to saccharides degradation produced upon treatment with isopropanol at 463 K. None of these traces were detected in either SeolA or B, probably due to the autohydrolysis step that removed the hemicellulose residue prior to soda ethanosolv extraction.

Quantitative diffusion ordered spectroscopy NMR (q-DOSY) relies on the quantitative ^1^H NMR that requires of using an internal standard [52,53] and permits reliable quantification when using stimulated-echo DOSY sequences. Q-DOSY presents problems with complex mixtures that could have proton overlapping [54] and thus different decays, hence making data fitting difficult [55]. In this case, semi-quantitative DOSY combined with ^31^P NMR of the reaction mixtures has been used to make an estimation of the purity of starting lignins and the poly-(hydroxy)-aromatic ether fractions in the as-mentioned reaction mixtures and to evaluate the degree of the ethanol side-reactions (see below). This estimation has been carried by means of the calculation of the percent of aromatic carbon (Table 2, SI.3, Equation (S1) and Equation (S2) in Supporting Information) that reflects the proportion of aromatic carbon atoms (i.e., from lignin) in the reaction mixtures.

Most of the diffusion traces in downstream biorefinery lignins corresponded either to partially degraded cellulose after enzymatic hydrolysis, to degradation products upon pretreatment or to acetyl moieties derived from derivatization. The low solubility of the samples did not allow to obtain reliable results from DOSY integration. Semi-q-DOSY could be tested in the determination of the purity of technical lignins, SeolA, B and OrgB. The purity of lignins can be estimated using the positive integration values of the aromatic (7.1–6.0 ppm) and aliphatic regions (3.4–2.6 ppm and 2.4–0.5 ppm). The aliphatic region in the range 4.2–3.5 ppm was excluded for this estimation because hydrogen atoms either in methoxy and β-O-4 motifs appear in this range, which can lead to data misinterpretation. DOSY integration refers to the number of hydrogen atoms. However, the H/C ratio in aromatic and aliphatic moieties is different (2:1 in aliphatic, 1:3 in syringyl units and 1:2 in guaiacyl units, see Supporting Information SI.3). Selected samples were also analyzed using ^31^P NMR after derivatization with 2-chloro-4,4,5,5-tetramethyl-1,3,2-dioxaphospholane (TMDP) to estimate the guaiacyl to syringyl ratio (G/S). This permitted a more accurate determination of H/C ratio for the aromatic compounds (See Supporting Information SI.3, Seq.1 and 2). The integration values of aromatics are shown in Table 2, resulting in 97% and 90% aromatic C for SeolA and OrgB respectively, that is consistent with the values showed in Table 2. 98% of aromatic carbon was measured for SeolB while its purity was 69%. The difference accounts for the content in ashes (ca. 30%) that are not detected by NMR.

HSQC and HSQC-TOCSY analyses of the lignins were consistent with DOSY. Technical lignins presented intense H-C cross-peaks in the aromatic regions. As expected, SeolA (see Figure S10) presented cross-peaks in the aromatic regions that corresponded to guaiacyl units together with signals corresponding to this guaiacyl units functionalized with double bonds. In the case of OrgB and SeolB (see Figures S11 and S12 in Supporting Information), cross-peaks corresponding to both guaiacyl and syringyl units could be detected in the aromatic region. H-C cross-peaks corresponding to aromatic units could not be found in acetylated BioA nor in acetylated BioB (see Figure S13 in the Supporting Information) due to their low solubility in DMSO-*d_6_*. The most important cross-peak in all samples corresponded to the methoxy groups in 3.7–55.0 ppm region. This cross-peak was accompanied with cross-peaks corresponding to H_α_-C_α_, H_β_-C_β_ and H_γ_-C_γ_ in β-O-4 structures together with H_γ_-C_γ_ in resinol and cinnamyl. In the case of BioA, some H-C cross peaks corresponding to the anomeric carbon of saccharides could be detected. This suggested that the detected signals in the range 3.0–5.0 ppm/ 60–100 ppm in the HSQC-TOCSY spectrum corresponded to H-C and long-distance correlations from partially degraded cellulose after incomplete enzymatic hydrolysis. Remarkably, no significant cross-peaks were found in the aliphatic region for SeolA or B, which suggests that degradation reactions did not occur during lignin isolation neither in the solvent nor in the saccharides, whereas in OrgB cross peaks corresponding to alkyl side chains were detected.

### 3.2. Characterization of the HTC-M Catalysts

The textural properties of the hydrotalcite supported Ni-Cu catalysts and of the parent calcined support HTC are shown in Table 3. The data showed that the surface area of the HTC sample was much higher than those of the HTC-M catalysts. This may be due to blocking of the smaller pores (micro and small mesopores) when adding the nitrate salts of the components onto the support, though some nitrate decomposition could take place during sample pretreatment prior to nitrogen adsorption measurements. All of the HTC-M catalysts showed low specific surface areas (~25–40 m^2^/g) and average pore diameters in the intermediate mesopore range (~15–30 nm), which could mean than nickel nitrate and copper nitrate were properly incorporated onto the catalysts [56]. The specific surface area and pore volume of the HTC-0 catalyst were the largest in all the catalysts series, while that of the HTC-5 was the smallest. A similar trend was observed for the pore volume.

As for the metal content of the catalysts, determined by ICP-OES, in general good correspondence between the nominal and the measured metal contents in the catalysts was found, which indicates that the incorporation of the nitrates onto the hydrotalcite structure during the impregnation step was correct.

Regarding the XRD analyses, representative diffraction patterns of the NiCu-HTC fresh and calcined samples –prepared with analytical purposes- can be checked in the Supplementary Materials section (see Figure S1). The patterns show that the samples present a high degree of crystallinity, with broad and in general short diffraction peaks, which qualitatively indicates that the size of the crystals is small. Most of the peaks in the fresh sample could be ascribed to the presence of the aluminum carbonate hydroxide hydrate crystalline phase derived from the hydrotalcite support. The calcined samples also had diffraction peaks that corresponded to CuO and NiO, though overlapping of the peaks of the standard diffraction patterns with those of their corresponding Ni and Cu aluminum carbonate hydroxide hydrates impeded to elucidate unambiguously the actual crystalline structure of the materials. It seems very likely that a combination of both oxides and hydroxides co-exists, given the relatively mild calcination conditions used, confirming the correct incorporation of the metals on the support.

### 3.3. Aromatics Formed after Lignin Depolymerization

Depolymerization of BioA and B was first tried by catalytic hydrogenolysis using Ru/C (data not shown), in ethanol at 523 K. However, this treatment was not efficient in downstream biorefinery lignins because of their high recalcitrance. Recalcitrance in downstream biorefinery lignins is higher than in native lignins after acidic thermo-chemical pretreatment due to the formation of C-C bonds between aromatic units that hinders the release of low molecular weight units. In addition, the strong acidic thermo-chemical pretreatment reduces the number of β-O-4 motifs in the lignin [42], preventing the nucleophilic attack of the ethanolic solvent to the α position of the β-O-4 motifs that triggers the hydrogenolysis reaction. Furthermore, both BioA and B were not soluble in ethanol, which caused diffusional limitations from the lignin into the catalyst.

Thus, the BCD reaction conditions for BioB were carefully optimized instead in aqueous medium [6]. Basic aqueous medium enhanced the solubility of lignin upon formation of phenolates that also initiates the depolymerization process. Amongst the different bases, aqueous NaOH was found to be effective at relatively low temperatures (473 K) and reaction times in the 240 min–360 min range (see Table 4), whereas ethanolic NaOH proved to be ineffective. Monomer weight yields (wt.%) under these conditions ranged between 9.1 wt.% and 9.7 wt.%, whereas longer reaction times provoked a decrease in monomer yields due to repolymerization. Guaiacol, 10, was the major monomer (30%), followed by syringol, 20, and phenol, 1 (25% and 18% respectively). BCD of BioA under the same conditions only produced 4.1 wt.% of monomeric aromatics. As expected, guaiacol, 10, was the major monomer (66%), followed by other guaiacol derivatives such as methylguaiacol, 11, ethylguaiacol, 12 and acetovanillone, 18 (ca. 6% each).

Given the excellent yields obtained with downstream biorefinery lignins, the optimized reaction conditions were tested in the BCD of OrgB at 463 K in H_2_O. Low monomer yields were obtained, 5.2 wt.%, which was due to the low solubility of OrgB in the reaction medium. Hence, the same reaction conditions were tested using ethanol as solvent, yielding 2.1 wt.% of aromatic monomers. Monomer distribution was different, though, being 1 (47%) the most abundant monomer when BCD was carried in ethanol and 20 (45%) when the reaction was carried in H_2_O. Noteworthy, in the case of aqueous BCD of OrgB, syringol derived monomers were predominant (ca. 69%) whereas in the case of ethanolic BCD, phenolic derivatives (60%) were predominant suggesting that, under these operating conditions, severe demethoxylation took place. Homogeneous BCD was finally carried in ethanol at 543 K to test the effect of reaction temperature, OrgB_543-EtOH_, but only 1.8 wt.% of aromatics was obtained, despite the high solubility of OrgB, accounting for severe repolymerization (see Table 4) due to harsh reaction conditions.

Thus, HTC-M catalysts were deemed a reasonable option to perform BCD on OrgB in ethanol. Besides the intrinsic advantages of using heterogeneous catalysts, HTC-Ni catalysts have already proved their efficiency in lignin BCD. Heterogeneous BCD of OrgB was tested in supercritical ethanol (543 K, 110 bar, see Table 4). Non-catalyzed depolymerization of OrgB under these conditions provided slightly higher monomer yields than NaOH ethanolic depolymerization at 543 K, 3.2 wt.%, that is presumably due to ethanolic fractionation of OrgB. In addition, catalytic depolymerization in scEtOH (543 K) of OrgB using the calcined HTC support (without impregnation) provided slightly lower aromatic monomers yields than in the blank reaction. It is worth mentioning that homosyringaldehyde, 26, (48%) was the most abundant monomer. Hydroxy-3-methoxyphenylacetone, 15, (26%) and phenol 1, (14%) yields were also relatively high. Monomer yield increased beyond 5 wt.% with HTC-0, HTC-2.5 and HTC-4, reaching its maximum with HTC-1, 6.8 wt.%, and decreased with the highest nickel content in the solid, HTC-5, 2.4 wt.%. Monomer distribution was somewhat stable at different Ni/Cu loadings though, being in all cases homosyringaldehyde 26 the most abundant aromatic monomer (44–56%) followed by 15 (15–20%). This suggest that, besides the basic activity of HTC-M, which is enhanced by nitrate anions as described by Kruger et. al. [44], using scEtOH as solvent promotes the oxidation of the alkyl side chains providing 15 and 26 as mayor aromatic monomers.

HTC/Ni-Cu catalyzed depolymerization of BioB in aqueous media provided negligible monomer yields due to mass transfer limitations as mentioned before for Ru/C. BCD of SeolA lignins using Ni and/or Cu catalysts supported on HTC proved to be unsuccessful. Monomer yields were in all cases under 3.0 wt.%. Almost 85% of the monomers under these conditions were phenol (85%) and syringyl monomers (15%).

As mentioned before, most of the studies on catalytic depolymerization of technical lignins rely on heterogeneous catalysis of hydrogenolysis reactions. Direct hydrogenolysis of technical lignins using commercially available catalysts was tried in ethanol because of its ability to prevent repolymerization [39,40]. SeolA was reacted in ethanol in the presence of Pd/C catalyst at 473 K, though monomer yields were under 3.0 wt.% at the selected operating conditions. Hydrogenolysis at 523 K under hydrogen in ethanolic medium in the presence of Ru/C, SeolA_Ru_, performed only slightly better (3.5 wt.%), being guaiacyl monomers (86%) the major monomers followed by phenols (3%). Among them, 4-ethylguaicol, 12, was the most abundant monomer followed by 4-propylguaicol, 13. It is worth noting that gigantol, 19, was present with ca. 6%. Hydrogenolysis of SeolB and OrgB under the same conditions provided slightly higher bioaromatics yields, 3.9 wt.% and 6.3 wt.% respectively. However, as could be expected, the monomers distribution changed, being the most relevant monomers homosyringaldehyde (20% and 37%), 26, and 4-hydroxy-3-methoxyphenylacetone (32% and 31%), 15 (see Table 5).

The alleged synergistic effect of Ru/C and basic catalysis is still a matter of controversy [17,41]. Therefore, homogeneous BCD and Ru/C hydrogenolysis were carried out in separate processes (see Scheme 2). Homogeneous BCD using NaOH seemed to be a plausible option. However, in view of the results obtained with OrgB, reaction conditions were slightly modified. The low solubility of SeolA in H_2_O was circumvented using NaOH in EtOH/H_2_O mixtures at lower temperatures, 493 K and 523 K, for 60 min, to obtain mixtures SeolA_493_ and A_523_, respectively, in almost quantitative yields. Low monomer yields were obtained under these conditions, 0.6 wt.% and 1.1 wt.%, respectively. The monomer distribution showed that, as expected, the major monomers were guaiacyl monomers (81% and 88%, respectively) followed by syringyl monomers (10% and 5%, respectively). As a thumbnail rule, syringyl monomers are not present in pine lignins; however, it has been reported that small amounts can be found in pine barks [57]. ^31^P NMR spectrum of derivatized SeolA confirmed the presence of syringyl units by the presence of a shoulder at 143.1 ppm. These syringyl units are more easily detached from lignin than guaiacyl units since the formation of C-C bonds occurs more easily in the latter. Nevertheless, both DOSY and SEC experiments (see below) evidenced that, although the monomer yield was relatively low, M_n_ and M_w_ in SeolA_493_ and A_523_ were much lower than in SeolA, which indicates that some depolymerization had occurred.

SeolA_493_ and A_523_ were subjected to hydrogenolysis in the presence of Ru/C in ethanol at 523 K, SeolA_493-Ru_ and A_523-Ru_, respectively. Monomer yields in SeolA_493-Ru_ steadily increased with reaction time from 2.4 wt.% (0 min) to 4.2 wt.% (after 120 min) and remained constant thereafter. Guaiacyl monomers were the most abundant (ca. 82%) again and, amongst them, 12 was the most abundant. However, the content in 12 decreased from 53% to 42% over reaction time (0 to 240 min), with a concomitant increase in guaiacol, 10, whose content was raised from 12% to 20%, whereas the proportions of 11 and 4-acetovanillone, 18, remained almost constant with reaction time (10% and 7% respectively). This suggests that increasing reaction times produced an increase in 10 due to alkyl side-chain breakage in 12. A noticeable increase in the aromatic monomer yields was observed when SeolA_523_ was subjected to hydrogenolysis under the same conditions, SeolA_523-Ru_. Consequently, the monomer yield increased from 5.0 wt.% at 0 min to 7.3 wt.% after 30 min and remained constant (6.7 wt.%) at longer reaction times. The proportion of guaiacyl monomers rose beyond 90% in all cases, being 12 the most abundant amongst them (ca. 45%) followed by 10 (ca. 30%). Noteworthy, any change in the monomer distribution in the reaction mixture could be found regardless of the reaction time. It can be observed that the relative amounts of 11 and 12 are very similar in SeolA_493-Ru_ and A_523-Ru_, but there is a strong increase in 10 (from 20% to 3%) together with a decrease in 18 (10% to 2%, at 30 min of reaction time), and gigantol, 19, (6% to 2%, at 30 min of reaction time).

Next, SeolB was reacted following the optimized procedure for SeolA. Monomer yields for SeolB_523_ (9.7 wt.%) were, however, much higher than for SeolA_523-Ru_, because of lower cross-linking in the starting lignin. The most abundant monomers were 4-hydroxy-3-methoxyphenylacetone (36%), 15, followed by syringol, 20 (17%). Further hydrogenolysis in the presence of Ru/C produced a slight increase in the aromatics yield up to 11.2 wt.% with 15 being the most abundant bioaromatic (45%) followed by 12 (16%) and 20 (15%). Finally, treatment of OrgB with NaOH at 523 K in EtOH/H_2_O, OrgB_523_, provided 7.5 wt.% yield to phenolic monomers, being 20 (34%) the most abundant followed by phenols, while 15 was not detected and 26 accounted for just 11% of the bioaromatics. Hydrogenation of OrgB_523_ also produced a slight increase in aromatic monomers, 8.7 wt.%, with 15 (36%) and 20 (19%) being the most abundant monomers. This suggested that most of the depolymerization in SeolB and OrgB occurred under BCD in EtOH/H_2_O at 523 K, while Ru/C catalyzed hydrogenolysis had little effect in monomer yields.

### 3.4. Poly-(Hydroxy)-Aromatic Fractions

**Figure 1:**
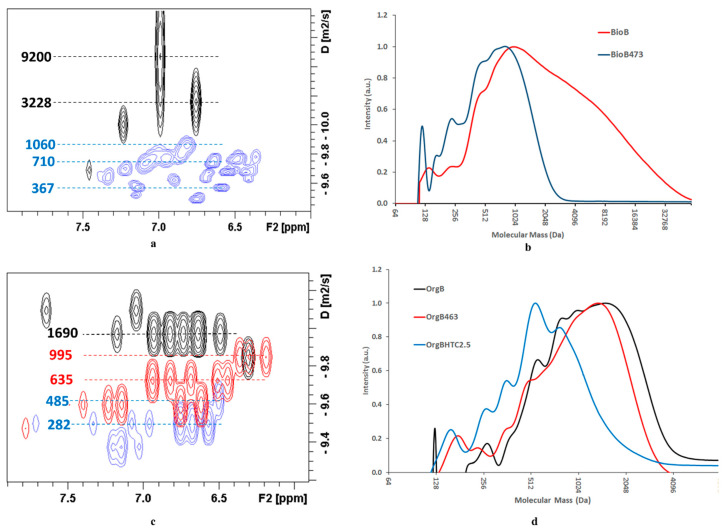
(**a**) Aromatic region for the DOSY spectra of BioB (black) and BioB_473_ (blue); (**b**) SEC for BioB (red) and BioB_473_ (blue); (**c**) DOSY spectra and (**d**) SEC for OrgB (black), OrgB_463_ (red) and OrgB_HTC2.5_ (blue). Figures in black, red and blue correspond to polystyrene, PS, calibration.

BioB was subjected to BCD in the presence of NaOH at 473 K, BioB_473_. DOSY spectra (see Figure 1a) clearly showed the difference in apparent mass between the aromatic hydrogen atoms in the starting lignin and those corresponding to the BCD reaction mixture, with fractions in the range of 360 Da–1060 Da. Moreover, some diffusion traces were detected in the aliphatic region (see Figure S5 in Supporting Information), which correspond to degradation products from the peeling reactions of saccharides in the basic medium [58]. A similar observation can be made when OrgB is reacted under similar reaction conditions at 463 K (see Figure 1c), OrgB_463_. The apparent mass decreased from 1454 Da to 709 Da (see Table 6) according to DOSY measurements in the aromatic region, although the SEC analyses did not show any noticeable change but a slight shift to lower masses (see Figure 1d). However, when the same reaction was carried out in ethanol under the same conditions, the mass distribution changed drastically and the size distribution narrowed with a prominent peak at ca. 530 Da, although the monomer yield decreased to 2.1 wt.% (see Table 4, Table 6 and Figure S9). The maximum monomer yield in the depolymerization of OrgB was obtained using HTC-1 in scEtOH (543 K), with an increase in the monomers yield up to 6.8 wt.%. M_n_ determined by SEC was 694 Da while the apparent mass determined by DOSY was 428 Da, which represents a noticeable decrease in apparent mass from the starting OrgB and B_463_ in H_2_O. Size distribution was clearly shifted to lower masses when BCD was catalyzed by HTC-M, OrgB_HTCx,_ whatever the Ni and/or Cu loading. In addition, DOSY measurements (see Figure 1) showed traces with noticeable lower apparent masses than OrgB_463_, which evidenced that depolymerization is much more extensive when the reaction is carried in ethanol than when using H_2_O as solvent, which can be due to the highest solubility of OrgB in ethanol than in H_2_O.

**Figure 2:**
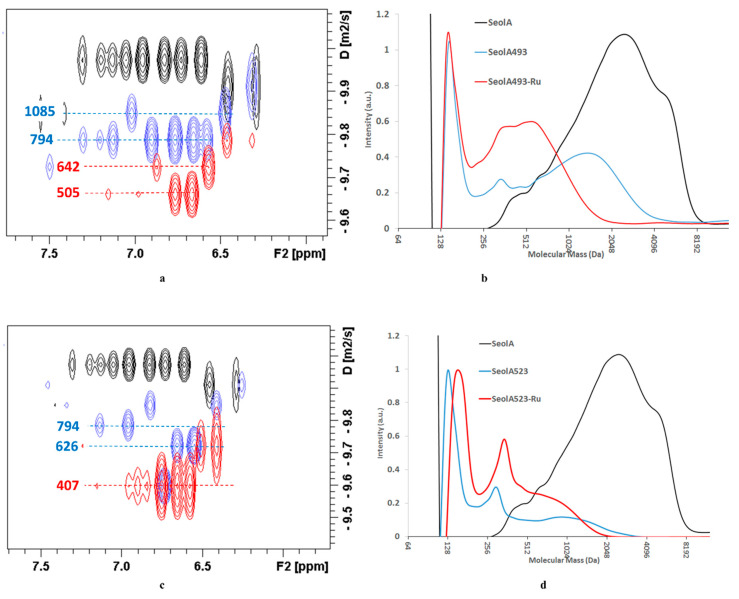
(**a**) DOSY in the aromatic region and (**b**) SEC for SeolA (black), A_493_ (blue) and A_493-Ru_ (red). (**c**) DOSY in the aromatic region and (**d**) SEC for SeolA (black), A_523_ (blue) and A_523-Ru_ (red). Figures in red and blue correspond to PS calibration.

SeolA and B depolymerization reactions were conducted using a different strategy that consisted of a two-step approach. Firstly, NaOH treatment in EtOH/H_2_O was carried out at 493 K or 523 K for 60 min runs, SeolA_493_ and A_523_. The monomer yields in both cases were low, 0.6 wt.% and 1.0 wt.%, respectively (see Table 5). However, DOSY for SeolA_493_ and A_523_ showed in both cases that depolymerization of SeolA did occur, being more extensive in SeolA_523_, 660 Da, than in SeolA_493_ 1039 Da (see Table 6). The most representative diffusion trace for SeolA_493_ was centred at 800 Da but representative traces were present in the 800–1600 Da range, whereas in the case of SeolA_523_, the most representative diffusion traces appeared centred at 630 Da with some small diffusion traces around 1024 Da (see Figure 2).

**Figure 3:**
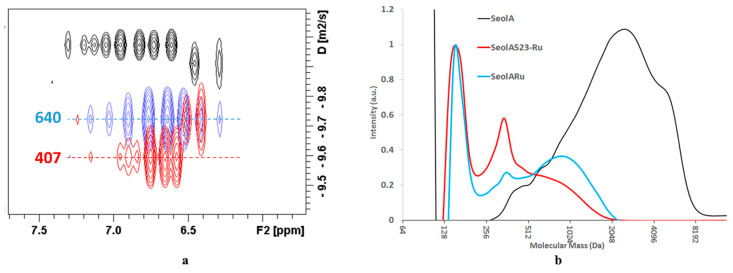
(**a**) DOSY spectra and (**b**) SEC for SeolA (black), A_523-Ru_ (red) and A_Ru_ (blue). Figures on the left correspond to PS calibration.

Hydrogenolysis of SeolA_493_ and A_523_ was done in scEtOH at 523 K in the presence of Ru/C (20 bar). As expected, the monomer yields were higher than in starting SeolA_493_ and A_523_, reaching 4.2 wt.% in SeolA_493-Ru_ and 7.3 wt.% in SeolA_523-Ru_. DOSY spectra showed in this case a more pronounced effect of the treatment in SeolA_493-Ru_, since its apparent mass decreased from 1039 Da to 691 Da (see Table 6). In the case of SeolA_523-Ru_ the apparent mass was similar to that of SeolA_523_ (ca. 660 Da) and, although DOSY spectra showed some slight differences between SeolA_523_ and A_523-Ru_, it can be appreciated that an important fraction of the diffusion in SeolA_523_ overlaps with those of SeolA_523-Ru_ (see Figure 2). Noteworthy, the estimated apparent masses, 746 Da, were only slightly higher when SeolA was directly treated with Ru/C at 523 K in ethanol and diffusion traces were essentially similar to those of SeolA_523-Ru_ (see Table 6 and Figure 3) although its chemical monomer yield was much lower.

**Figure 4:**
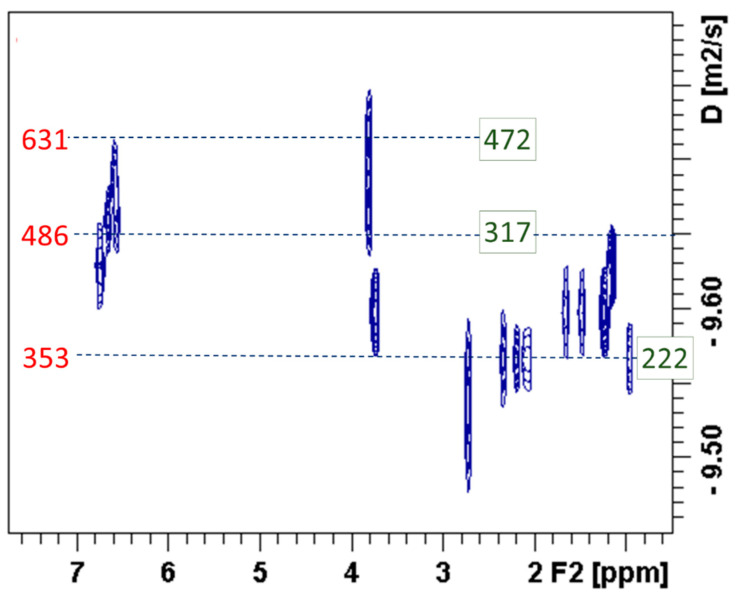
DOSY spectrum for SeolA523-Ru. Figures on the left correspond to PS calibration and figures on the right to PEG calibration. Red dotted squares correspond to the integration regions in the aromatic and in the aliphatic regions.

Data provided by DOSY spectroscopy shed light about the somewhat strange SEC chromatograms obtained after depolymerization of SeolA (see Figure 2 and Figure 3). Apparent masses determined by DOSY do not seem to match with the most prominent peaks determined by SEC. Thus, in all analysed samples, a peak at ca. 130 Da appeared. This peak is the most intense in the samples treated with Ru/C in EtOH, but it is also important in OrgB samples depolymerized using HTC in EtOH. This is consistent with the detection of diffusion traces in the aliphatic region of the DOSY spectra that might correspond to compounds arising from ethanol via hydrogen transfer mechanism (see Figure 4 and Figure S7).

Besides this peak, the rest of SEC chromatograms presented a broad distribution which is centred at similar mass values than those estimated by DOSY for the aromatic region (i.e., poly-(hydroxy)-aromatic fraction). In the case of SeolA_493_ and A_493-Ru_, broad mass fractions were present, which were more important in SeolA_493,_ centred at 1360 Da, and at 570 Da in SeolA_493-Ru._ In SeolA_523_, A_523-Ru_ and A_Ru_ mass distributions were much narrower and presented a prominent peak at ca. 360 Da (see Figure 2). OrgB_HTC_ presented also a broad distribution with two major peaks centred at 526 Da and 758 Da that may correspond to three and four-unit aromatic compounds.

It is important to note that in all depolymerization reactions of OrgB, carried out in EtOH in the presence of either NaOH or HTCs with different Ni-Cu loadings, no significant differences in M_n_ (ca. 700 Da) could be found among the different Ni-Cu loadings or in the blank reaction. However, when BCD was carried out in H_2_O in the presence of NaOH, M_n_ was much higher, ca. 1200 Da, which suggests that fractionation of lignin is caused by the ethanolic solvent at high temperatures (viz. thermos-solvolysis). Similarly, direct hydrogenolysis in the presence of Ru/C in ethanol and BCD in ethanol/H2O at 523 K of SeolA (see Figure 3), B and OrgB, M_n_ was in the range of 600–700 Da, that also suggest the essential role of thermos-solvolysis in lignin depolymerization.

Diffusion traces in the range of 300–330 Da using the PS calibration curve or in the range of 185–300 Da using PEG curves, could be detected in all the DOSY spectra. This agrees with the peak that can be observed in most SEC chromatograms in the range of 275–350 Da. These diffusion traces in the aromatic region can be attributed to the presence of dimers (e.g., guaiacylglycerol-β-guaiacyl ether, gigantol, etc.). It must be highlighted that in all the studied samples derived from technical lignins, SeolA, B and OrgB, diffusion traces in this apparent mass range could be detected in the aromatic region, which suggests that most of this peak may come from the presence of two-unit aromatic compounds. However (see Figure 4 and below), diffusion traces corresponding to these relatively low apparent masses were much more intense in the aliphatic regions.

DOSY spectra presented in most cases a remarkable difference between the diffusion coefficients associated to the aromatic and the aliphatic regions, being the latter significantly higher. Diffusion traces in the DOSY spectra (see Figure 4) associated to the aromatic region are related to apparent masses above 400 Da, and centered in the range of 486–630 Da, whereas the aliphatic regions are below 500 Da, according to PS calibration, and below 300 Da according to PEG calibration (for the comparison of average apparent masses in both regions see Table 6). In a previous work [6], these traces in the aliphatic region were associated to the peeling reaction of saccharide impurities in the starting lignins, as is the case for BioA and B. However, diffusion traces at low apparent masses were also observed in the depolymerization of technical lignins, as it can be clearly seen for SeolA_523-Ru_ (see Figure 4). GC-MS analysis from the depolymerization reactions carried in SeolA, B and OrgB presented products whose origin can be associated to side reactions from ethanol (viz. hexanol, 2-ethyl-butan-1-ol, 4-methyltetrahydro-2*H*-pyran-2-one, etc.). Both Ru/C and Cu-containing HTC catalysts are able to promote hydrogen transfer reactions from ethanol that prompt the Guerbet reaction [47,59] that produces long chain alcohols. Given the reaction conditions, it can be expected that higher mass ethanol derivatives, that were not detected by GC-MS were produced.

Semi-q-DOSY was used in Section 3.1 to estimate the purity of isolated lignin. A similar approach was used to estimate the percent of C atoms that correspond to aromatic rings in the reaction mixtures (see Table 6). The percentage of aromatic C is relatively low in the depolymerization of BioA and B (54% and 58%) because of the peeling reaction of partially degraded saccharides in the starting lignins. OrgB_463_ (73%) presents higher aromatic C content than BioA_473_ and B_473_ because no degradation of saccharides occurred upon BCD.

SeolA_Ru_, B_Ru_ and OrgB_Ru_ also presented relatively low contents in aromatic C (65%, 71% and 68% respectively) according to DOSY integration. However, in this case the decrease in the aromatic percentage of C atoms accounts for the formation of aliphatic compounds upon reaction of ethanol at high temperatures, following the hydrogen transfer mechanism in a similar fashion than in the Guerbet reaction. The estimated aromatic C is higher in SeolA_493_ and A_523_ (83% and 66%) than in SeolA_493-Ru_ and A_523-Ru_ (61% and 56%) respectively that is due to more extensive ethanol side reaction upon reaction at 523 K. A similar trend was observed in SeolA_523_ and A_523-Ru_ (66% and 56%) and OrgB_523_ and B_523-Ru_ (61% and 44%).

This effect was more pronounced when OrgB reacted with NaOH. 73% and 62% of aromatic C were estimated when the reaction was run at 463 K in H_2_O and ethanol respectively, whereas only 16% was estimated when the reaction was run in ethanol at 543 K. Even more interesting, when the BCD was carried out using HTCs at 543 K, the estimated aromatic carbon increased with increasing nickel contents. Thus, 38% of aromatic C was estimated for treatments with HTC-0 and HTC-1, 44% for HTC-2.5, 49% for HTC-4 and 57% for HTC-5. This percentage of aromatic carbons was low, suggesting that the Guerbet reaction took place to a great extent, which is consistent with previous reports that described that the Guerbet reaction is catalyzed by HTC-Cu catalysts [47].

^31^P NMR analyses after derivatization of some reaction mixtures provided the number of aromatic hydroxy groups per gram of sample (see Table 6). This can be also a good parameter to evaluate the purity of the mixture in terms of poly-(hydroxy)-aromatic content. Indeed, this parameter followed the same trend that the aromatic C content in comparable reaction mixtures. In the case of the depolymerization of OrgB it can be observed that OrgB_463_ carried out in H_2_O presented 5.09 mmol aromatic OH/g while the same reaction in ethanolic medium provided a reaction mixture with 2.20 mmol aromatic OH/g. Similarly, OrgB_HTC2.5_ contained 2.61 mmol aromatic OH/g. In the reaction mixtures of the SeolA series it can be also observed that the content of aromatic OH/g decreased from SeolA_493_ to A_493-Ru_ and from SeolA_523_ to A_523-Ru_, which can be attributed to the increase in aliphatic carbon derived from side reactions produced by the presence of ethanol.

Some of the diffusion traces detected in the aliphatic region may also come from C-and O-alkylation under reaction conditions with scEtOH, as it has been previously described [39,47,60]. HSQC of SeolA_523_ and A_523-Ru_ in the aliphatic region (see Figure S14 in Supporting Information) presented cross-peaks that are compatible with alkylated aromatic compounds. A similar observation can be made for OrgB_HTC2.5_ (see Figure S15 in Supporting Information), but not in those reactions carried in H_2_O. However, the intensity of these signals was relatively low and was only detected after selective peak-picking treatment of the raw spectrum, and their diffusion coefficient was slightly higher than that of the corresponding aromatic region. Finally, O-alkylated and C-alkylated (others than gathered in Table 4 and Table 5) products were not detected by GC-MS.

## 4. Discussion

The results shown above reveal that BCD in the aqueous medium is still a valuable option for the depolymerization of highly recalcitrant lignins, because the basic media promotes the formation of phenolate anions that increase the solubility of the lignin, which evolve to quinone methides initiating the process. It must be noted that BioA and B were obtained after acidic thermochemical treatment of poplar and pine chips. This acidic pretreatment promotes the formation of C–C bonds between the aromatic units that increased the recalcitrance of the lignin, which explains their low solubility in organic solvents [42]. Organosolv-derived lignin from poplar sawdust, OrgB, was poorly solubilized in aqueous medium, hence yielding low amounts of depolymerization products in NaOH BCD, whereas the opposite situation took place in ethanolic medium, which not only solubilized OrgB lignin effectively but also acted as reactant causing thermo-solvolysis. BCD reaction conditions for OrgB in ethanol were harsh that provoked repolymerization and, therefore, low monomer yields.

As concerns heterogeneously catalyzed depolymerization of lignins using Ni-, Cu- and NiCu-HTC catalysts, the results show that the yield to aromatics only increased slightly in comparison to homogeneous BCD. The catalysts primarily exerted a role in modifying the selectivity to the different products. Moreover, impregnating the HTC supports with Ni and/or Cu also caused activity and selectivity to vary, and synergistic effects were observed in the simultaneous use of Ni and Cu as active metals. Nonetheless, the formulation of bimetallic NiCu-HTC-s must be carefully done. In fact, the increase in monomers containing carbonyl groups (15 and 26) taking place upon depolymerization using the different HTC-M catalysts prepared can be associated with the decrease in copper content. These compounds are more prone to repolymerization reaction under basic conditions that can be in the origin of the decrease in the overall monomer yield at the lowest copper loadings.

One important feature in HTC/Ni-Cu catalysts is that, in the opposite way to BCD using NaOH, monomers with side alkyl chains are produced with noticeable amounts of alkyl-guaiacols, 12–13 and 15 and alkyl-syringols, 26, which are hardly detected in NaOH depolymerization of BioB and OrgB in aqueous medium. This difference can be due either to the depolymerization mechanism itself or to the C-alkylation that may occur after depolymerization, as it has been already described in the presence of Cu-Mg-Al mixed oxides as reported by Huang et al. [47]. Concerning the depolymerization mechanism, in the case of Ni-HTC, Kruger et al. [44] suggested that the nitrate ion has an important role. Under the reaction temperature used in this study, nitrates can be decomposed to NO and NO_2_ that would promote lignin nitration that may accelerate the β-O-4 cleavage. In the same study, it was found vinylphenol, from decarboxylation of *p*-coumaric acid, as the major monomer in the Ni-HTC depolymerization of lignin. In addition, as mentioned before, Ni-modified catalysts present strong binding sites for ether linkages and specifically cleave the C–O linkages, yielding side alkyl chains in the presence of hydrogen [45], which in this case would be provided by the dehydrogenation of ethanol that is in the origin of the Guerbet reaction (see below) [59]. Concerning C- and O- alkylation, the Guerbet reaction and alkylation using HTC-Cu catalysts have also been described by Huang et al. [47]. In some cases, methylation of the aromatic ring was also observed when using methanol as solvent medium and HTC-Cu catalysts [61]. In any case, neither O-alkylated phenols nor other aromatics than those gathered in Table 4 and Table 5 were detected by GC-MS in the present study. Although the as-used reaction conditions are more similar to those described by Kruger et al. than those described by Huang et al. (543 K vs. 613 K and 653 K), the relatively high contents of homosyringaldehyde and ethylguaiacol in the HTC-catalyzed reaction do not allow us to totally discard C-alkylation.

The Guerbet reaction consists of a sequence of reactions that starts with metal-catalyzed dehydrogenation followed by aldol-condensation, dehydration and metal-catalyzed hydrogenation, which can lead to long chain alcohols under our reaction conditions. Indeed, as can be inferred from the DOSY and HSQC analyses presented above, the extent of the Guerbet reaction when using the Ni-, Cu- and NiCu-HTC catalysts is very large, which is in agreement with previous works [59].

As stated in the introduction section, other authors studied depolymerization of lignin-rich biorefinery streams derived from enzymatic hydrolysis of corn stover using Ni-HTC catalysts [44]. Despite selecting milder operating conditions in the present study, similar M_n_ values were attained, and monomer yields were similar than those presented by Kruger et al. [44]. However, it seems likely that in that study neither saccharide degradation reactions nor self-condensation reactions of the alcohol used as reaction medium were considered, which meant that monomer yields were lower than expected from SEC. Most of the fractionation attained is likely caused by the synergistic effect of the solvent and the HTC solids. It is interesting that the role of Ni in the depolymerization is somewhat less effective than the catalytic role of nitrates, which differs with the observations found in Sturgeon et al. [43]. However, it must be noted that the yields to monomers were not reported in that work. It could be possible that the decrease in M_n_ noticed in their SEC analyses would be mainly caused by thermal cracking reactions. In fact, the prevailing monomer was guaiacylketone, which seems logical if thermal cracking is the prevailing depolymerization route.

The results obtained using the Ru/C commercial catalyst showed that depolymerization of technical lignins obtained from pine or poplar wood was moderate in terms of activity, and that the noble metal catalyst could not outperform the HTC-M catalysts in OrgB. It is worth noting, however, that bio-aromatic based compound yield for OrgB was slightly higher than in SeolA and B, which can be attributed to a milder process upon its isolation (i.e., autohydrolysis step was not carried for OrgB). Notwithstanding that cleavage of alkyl side-chain bonds readily took place, C–C double bonds and carbonyl groups are more extensively reduced in the presence of this catalyst. Moreover, a number of aliphatic compounds as octanol, hexanol or 4-methyltetrahydro-2*H*-pyran-2-one were also detected by GC-MS, which suggests that hydrogenolysis proceeded via hydrogen transfer from ethanol, as it has been previously described for lignin depolymerization with Ru/C [62,63] or molybdenum carbide [64]. The formation of these byproducts may follow a similar mechanism to that described for HTC-catalyzed reactions (see above). Indeed, the proposed mechanism for Ru catalyzed hydrogenolysis in MeOH at 493 K indicates that the plausible pathway for Ru/C-catalyzed hydrogenolysis of lignin leading to C3 and C2-fragmented monophenols starts from a nucleophilic attack of MeOH on the benzylic position of β-O-4 units of lignin to give α-OMe β-O-4 structures and formaldehyde. Ru/C catalysts play an essential role on the regeneration of MeOH from formaldehyde and on the hydrogenation of the alkenyl side chains [17]. In fact, when the reaction was run under nitrogen atmosphere, no significant differences were found in comparison to the homologous run carried out under hydrogen atmosphere, which confirmed that the main reaction mechanism is hydrogen transfer from ethanol catalyzed by ruthenium.

Furthermore, it has been described that using Ru/C in the presence of a base causes a decrease in the content of monomer after lignin depolymerization [17]. This justified the decision adopted in this study of splitting hydrogenolysis in basic media into two separate steps for technical lignins: BCD in EtOH/H_2_O and Ru/C catalyzed hydrogenolysis. The different nature of the lignins is in the origin of the higher aromatic monomer yields for OrgB and SeolB (7–10%) compared to SeolA (1.1%) upon treatment with NaOH in EtOH/H_2_O. Further treatment with Ru/C, however, caused a noticeable increase in phenolic monomers yields in SeolA_493_ andA_523_ than in SeolB_523_ and OrgB_523_. Thus, as a thumbnail rule, monomer yields were lower in pine-derived lignins than in poplar derived lignins. Softwood lignin has a more condensed structure than hardwood lignin, because G subunits have one less methoxy group (in comparison with S subunits) which facilitates the formation of C–C bonds (such as 5-5 and β-5) and hinders depolymerization.

In addition, as already evidenced in our previous work [6], DOSY NMR, has proved to be a very valuable tool to estimate the actual apparent mass of the poly-(hydroxy)-aromatic fraction of lignins and their structural composition. Reaction mixtures after lignin depolymerization consist of a mixture of monomeric phenols and poly-(hydroxy)-aromatic ethers. The application of this latter fraction of poly-(hydroxy)-aromatic ethers is strongly dependent on their average or number molecular weight, M_w_ or M_n_ respectively. However, the estimation of the apparent masses using the diffusion coefficient, D, is not trivial because D is strongly dependent, among other factors, on the viscosity of the medium and the solvent-diffusate interactions. Interestingly, it could be observed that, in the estimation of poly-(hydroxy)-aromatic ethers with relatively high apparent masses, correlation with the values estimated by SEC were better using PS curves whereas in the case of low apparent masses this correlation was better using PEG curve. The reason lies in the different diffusate-diffusate and diffusate-solvent interactions that arise from the increasing hydrophobicity of the highest mass fractions that arise from and increasing importance of dispersion forces as the mass increases. Although some small discrepancies can be found between the apparent masses for the aromatic region, which corresponds to the apparent mass of poly-(hydroxy)-aromatic fraction, and M_n_, values are in good agreement.

Finally, semi-q-DOSY combined with ^31^P has been used for estimating the percent of aromatic carbon atoms in the reaction mixtures. Although this is not a quantitative measure, this has allowed for approximately determining the content of poly-(hydroxy)-aromatics in the reaction mixtures and evaluating the extent of ethanol side reactions (viz. transfer hydrogenation or the Guerbet reaction). In this case, this content decreases in the reactions that were carried out in ethanol and, amongst them, in those conducted with the harshest conditions because of the side reactions of the solvent.

## 5. Conclusions

BioA and B downstream biorefinery lignins were obtained from *P. radiata and Populus* Sp., respectively. SeolA and B were obtained after autohydrolysis and soda ethanosolv of *P. pinaster* and waste poplar sawdust, while OrgB was obtained from the latter via organosolv in isopropanol.

It has been shown that there is not a single solution to lignin depolymerization. Therefore, catalyst and reaction conditions must be carefully selected depending on the feedstock and the lignin isolation method.

BioA and B could only be depolymerized by NaOH BCD in aqueous medium. However, the best aromatic monomer yields in the depolymerization of technical lignins were achieved by BCD in ethanol/H_2_O followed by Ru/C catalyzed hydrogenolysis in ethanol. The catalyst plays an essential role in the selectivity. Ni-, Cu- and bimetallic NiCu-catalysts supported on calcined hydrotalcite produced slightly lower aromatic monomer yields in the heterogeneous BCD of OrgB in scEtOH, although, the synergistic effect of Ni and Cu caused a change in the selectivity yielding monomers with alkyl side chains bearing carbonyl groups.

The solvent and the reaction temperature are also key in the outcome of the depolymerization reaction. On one hand, the resulting poly-(hydroxy)-aromatic ether fractions presented similar apparent masses and SEC profiles when the depolymerization reactions were carried in a given solvent at a given temperature regardless of the catalyst used, suggesting that thermo-solvolysis is an essential part in lignin depolymerization. On the other hand, when the reaction was carried out in ethanol at high temperatures, a series of compounds derived from the hydrogen transfer mechanism in Ru/C catalyzed reactions and the Guerbet reaction in HTC-M catalyzed reaction were detected.

DOSY-NMR has proven to be a useful and reliable tool in selectively determining the apparent mass of the different fractions in the reaction mixtures. Semi-q-DOSY combined with ^31^P has been used for the first time in the estimation of the purity, not only of lignins, but also of the poly-(hydroxy)-aromatic fractions arising from lignin depolymerization and it has been essential to estimate the extent of the solvent side reactions.

## Figures and Tables

**Table 1:** Comparison of various lignin isolation methods. Adapted from reference [12].

Isolation Method	Lignin Name	Typical Process	Characteristics
Klason method [13]	Klason	2% H_2_SO_4_	Extensive structure change, hardwood lignin is partly dissolved
Kraft process [4]	Kraft	Na_2_S/NaOH	Highly modified, partially fragmented. High S content
Sulfite pulping process	Lignosulfonate	Extract lignin from waste liquor of the sulfate pulping process of soft wood.	Highly modified, high average molecular weights, cleavage of ether linkages, loss of methoxyl groups and formation of new C–C bonds
Björkman process [14,15]	Milled Wood Lignin (MWL)	Ball milling, then extracted by aqueous dioxane.	Similar to the native structure, possible depolymerization due to extensive milling
Organosolv process	Organosolv [9,10]	Using organic solvents to extract lignin.	Mild conditions, results in more unaltered lignin, solvent could be recovered by distillation
	Soda ethanosolv [11]	Basic reactant (NaOH, KOH…)	
Reductive catalytic fractionation [16,17]		Pd/C, Ru/C, Ni/Al_2_O_3_	Phenolic monomers, low molecular weight poly(-hydroxy)-aromatic ethers
Alkaline wet oxidation [18]	Alkaline wet oxidation	Oxidative pretreatment under alkaline conditions.	Partial degradation via β-O-4 cleavage
Steam explosion process [19]	Steam explosion lignin	High temperature steam explosion of the fibers.	Require little or no chemical input, short treatment time, low energy requirement, changes of certain functional groups
Mechano-catalytic process [20]	Mechano-catalytic lignin	Mechano- catalytic depolymerization.	Sulfur-free lignin

**Table 2:** Characterization of the different lignins.

	BioA	BioB	OrgB	SeolA	SeolB
Lignin (wt.%)	69.1	51.1	96.1	98	68.7
M_w_ (Da) ^a^	2950	5216	1636	3005	1806
M_n_ (Da) ^a^	435	514	1230	2209	1295
Dispersity	6.8	10.1	1.3	1.4	1.4
Apparent mass (Da) ^b^	1274 ^c^	7240 ^c^	1445	2122 ^c^	1477
S/G ratio ^d^	0.1	1.5	1.32 ^d^	0.15 ^d^	1.23 ^d^
Aromatic C (%) ^b^	n.m.	n.m.	90	97	69
Sulfur content (wt.%)	0.33	0.74	-	-	-
Glycan (wt.%)	23.2	38.8	0.4	0	0
Xylan-mannan (wt.%)	0.8	n.m.	n.m.	n.m.	0.8

^a^ Estimated using SEC; M_n:_ Number molecular weight; M_w_: Average molecular weight; ^b^ estimated using DOSY NMR; ^c^ Graphical estimation; ^d^ Calculated using ^31^P NMR (see Table S3 for details). S/G: syringyl to guaiacyl ratio n.m.: not measured.

**Table 3:** Textural properties of the HTC-M catalysts.

Catalyst	Ni–loading (wt.%)		Cu–loading (wt.%)				
	Reference	Measured	Reference	Measured	S_BET_^a^ (m^2^/g)	V_p_^b^ (cm^3^/g)	d_p_^c^ (nm)
**HTC**	0.0	0.0	0.0	0.0	150	0.17	3.8
**HTC-0**	0.0	0.0	5.0	5.6	39	0.35	31.5
**HTC-1**	1.0	1.0	4.0	4.4	34	0.17	18.5
**HTC-2.5**	2.5	2.6	2.5	2.7	36	0.17	17.4
**HTC-4**	4.0	n.m.	1.0	n.m.	37	0.21	21.9
**HTC-5**	5.0	4.9	0.0	0.0	24	0.13	22.3

^a^ Surface area ^b^ Pore volume ^c^ Average pore diameter.

**Table 4:** Distribution and yields (Y%) of phenolic monomers ^a^ from different base catalysts after BCD and heterogeneously catalyzed depolymerization of BioA, BioB and OrgB.

Lignin	Cat. ^b^	Solvent	T (K)	Y%	1	2	3	4–5	6	7	8	10	11	12	13	14	15	18	20	21	24	26
BioA	NaOH	H_2_O ^c^	473	4.7	3	-	2	3	-	-	-	66	5	6	-	6	-	6	2	-	-	-
BioB	NaOH	H_2_O ^c,e^	473	9.1	18	-	0	3	-	-	-	30	2	4	-	-	-	5	25	-	3	-
BioB	NaOH	H_2_O ^d^	473	9.7	19	-	0	0	-	-	-	31	2	4	-	-	-	6	28	-	3	-
OrgB	NaOH	H_2_O ^e^	463	5.2	24	-	-	-	-	-	-	-	-	-	-	-	6	-	45	1	23	-
OrgB	NaOH	EtOH ^e^	463	2.1	47	-	-	-	7	-	6	-	-	-	-	-	14	-	-	12	15	-
OrgB	-	EtOH ^e,f^	543	3.2	6	-	-	-	-	-	1	-	-	6	8	-	34	-	-	19	-	23
OrgB	NaOH	EtOH ^e^	543	1.8	65	15	-	-	-	-	-	-	-	1	3	-	2	-	2	-	-	-
OrgB	HTC	EtOH ^e^	543	2.4	14	-	-	-	-	-	-	-	-	8	4	-	26	-	-	-	0	48
OrgB	HTC-0	EtOH ^e,f^	543	5.4	11	2	-	1	-	-	-	-	9	13	-	-	15	-	-	-	-	47
OrgB	HTC-1	EtOH ^e,f^	543	6.8	9	-	-	3	-	-	-	1	-	7	11	-	15	-	-	10	-	44
OrgB	HTC-2.5	EtOH ^e,f^	543	5.4	8	-	-	0	-	-	-	-	-	7	14	-	19	-	-	1	-	49
OrgB	HTC-4	EtOH ^e^	543	5.0	8	-	-	-	-	-	-	-	-	8	17	-	19	-	-	-	-	48
OrgB	HTC-5	EtOH ^e^	543	2.4	8	-	-	-	-	-	-	-	-	8	6	-	21	-	-	1	-	56

^a^ (1) Phenol; (2) 2-ethylphenol; (3) o-cresol; (4–5) m-cresol/p-cresol; (6) Catechol; (7) 4-methylcatechol; (8) 4-ethylcatechol; (10) Guaiacol; (11) 4-methylguaiacol; (12) 4-ethylguaiacol; (13) 4-Propylguaiacol; (14) Vanillin; (15) 4-hydroxy-3-methoxyphenylacetone; (16) Homovanillyl alcohol; (17) Homovanillic acid; (18) Acetovanillone; (20) 2,6 dimethoxyphenol (Syringol); (21) 4-methylsyringol; (24) Syringaldehyde; (25) Acetosyringone; (26) Homosyringaldehyde; ^b^ Cat.: Catalyst; ^c^ reaction time 240 min; ^d^ reaction time 360 min; ^e^ reaction time 60 min; ^f^ reactions were made by triplicate.

**Table 5:** Distribution and yields (Y%) of monomers ^a^ from different depolymerization experiments carried at 523 K.

Lignin	Cat ^b^	Solvent	t (Min)	Y (wt.%).	1–9	10	11	12	13	15	16	17	18	19	20	21	24	25	26
**SeolA ^c^**	NaOH	EtOH/H_2_O	60	0.6	-	18	3	18	0	1	13	13	14	0	-	-	5	5	-
**SeolA_493_**	Ru/C	EtOH	0	2.4	-	12	10	54	5	-	-	-	7	5	-	-	4	0	-
			30 ^d^	2.2	4	9	10	42	4	1	-	-	11	6	-	-	7	2	-
			60 ^d^	3.7	-	17	11	46	5	-	-	-	7	6	-	-	4	-	-
			120 ^d^	4.2	2	19	11	36	3	3	-	-	9	10	-	-	3	-	-
			240 ^d^	4.2	-	20	10	43	3	1	-	-	6	8	-	-	1	2	-
**SeolA**	NaOH	EtOH/H_2_O	60 ^d^	1.1	1	45	8	29	3	0	-	-	5	2	-	-	-	5	-
**SeolA_523_**	Ru/C	EtOH	0	5	-	30	12	46	4	0	-	-	2	4	-	-	0	0	-
			30 ^d^	7.3	3	31	8	43	4	0	-	-	2	2	-	-	-	4	-
			60	5.1	-	32	11	44	4	2	-	-	-	0	-	-	-	-	-
			120	6.7	-	33	11	42	4	0	-	-	3	4	-	-	0	0	-
			240 ^d^	6.6	-	32	12	44	4	-	-	-	2	4	-	-	-	0	-
**SeolB**	NaOH	EtOH/H_2_O	60	9.7	2	11	3	14	9	36	-	-	-	-	17	3	-	1	3
**SeolB_523_**	Ru/C	EtOH	30	11.2	1	7	3	16	1	45	-	-	-	-	15	8	-	2	1
**OrgB**	NaOH	EtOH/H_2_O	60	7.5	27	7	0	12	0	-	-	-	-	-	34	9	-	-	11
**OrgB_523_**	Ru/C	EtOH	30	8.7	8	5	3	9	1	36	-	-	-	-	19	7	-	7	2
**SeolA**	Ru/C	EtOH	30 ^d^	3.5	2	7	8	51	15	1	-	-	5	6	-	-	-	-	-
**SeolB**	Ru/C	EtOH	30	3.9	-	-	-	11	4	32	-	-	-	-	14	11	-	5	20
**OrgB**	Ru/C	EtOH	30	6.3	5	4	1	-	8	31	-	-	-	-	-	-	5	-	37

^a^ (1) Phenol; (2) 2-ethylphenol; (3) o-cresol; (4-5) m-cresol/p-cresol; (6) Catechol; (7) 4-methylcatechol; (8) 4-ethylcatechol; (10) Guaiacol; (11) 4-methylguaiacol; (12) 4-ethylguaiacol; (13) 4-Propylguaiacol; (14) Vanillin; (15) 4-hydroxy-3-methoxyphenylacetone; (16) Homovanillyl alcohol; (17) Homovanillic acid; (18) Acetovanillone; (19) Gigantol; (20) 2,6 dimethoxyphenol (Syringol); (21) 4-methylsyringol; (24) Syringaldehyde; (25) Acetosyringone; (26) Homosyringaldehyde. ^b^ Cat.: catalyst; ^c^ reaction carried at 493 K; ^d^ reactions were made by triplicate.

**Table 6:** Apparent masses estimated by SEC and NMR DOSY for the starting lignins and samples.

	SEC		DOSY NMR				
Sample	M_w_ (Da)	M_n_ (Da)	Aromatic Region (Da)	Aliphatic Regions (Da)		Aromatic-OH (mmol/g)	Aromatic-C (%)
**BioB_473_**	1051	719	658	482	418	3.30	58
**BioA_473_**	1173	720	764	494	520	n.m.	54
**SeolA**	3005	2296	2200	279	553	4.57	97
**SeolA_493_**	1798	891	1039	285	463	3.58	83
**SeolA_493-Ru_**	811	523	691	441	502	2.97	61
**SeolA_523_**	1250	587	660	322	401	4.51	66
**SeolA_523-Ru_**	818	468	665	433	540	3.74	56
**SeolA_Ru_**	956	752	746	562	509	2.77	65
**SeolB**	1791	1206	1477	869	n.m.	1.69	98
**SeolB_523_**	1794	1194	742	492	447	2.54	68
**SeolB_523-Ru_**	1464	962	671	406	527	2.11	51
**SeolB_Ru_**	1434	980	720	539	425	n.m.	71
**OrgB**	1636	1230	1454	548	950	4.72	90
**OrgB (EtOH)**	1650	1195	1529	286	764	n.m.	85
**OrgB_463_**	1578	1186	709	245	515	5.10	73
**OrgB_463-EtOH_**	948	702	770	414	480	2.20	62
**OrgB_543-EtOH_**	1515	924	267	191	273	n.m.	16
**OrgB_EtOH-543_**	920	668	723	365	605	n.m.	65
**OrgB_HTC0_**	1328	724	420	348	421	n.m.	38
**OrgB_HTC1_**	908	694	428	396	459	n.m.	38
**OrgB_HTC2.5_**	821	6478	583	381	462	2.61	44
**OrgB_HTC4_**	1003	725	437	406	396	nm	49
**OrgB_HTC5_**	968	681	478	276	381	nm	57
**OrgB_523_**	1163	764	601	320	450	n.m.	61
**OrgB_523-Ru_**	836	593	638	319	457	n.m.	44
**OrgB_Ru_**	888	682	559	316	559	n.m.	68

## Supplementary Materials

The following are available online at https://www.mdpi.com/2218-273X/10/9/1338/s1, SI. 1 XRD analyses; Figure S1. Representative XRD diffraction patterns of the fresh and calcined NiCu-HTC samples; SI. 2 Chemical characterization lignin and reaction mixtures analysis; Table S1. Aromatic monomers and retention times; SI. 3. Experimental details for NMR characterization; Table S2 Integration regions used in the determination of averaged diffusion coefficients. Table S3. Integration regions used in the determination of aromatic –OH contents and G/S ratio; SI. 4. SEC and DOSY spectra; Figure S2. Normalized SEC chromatograms for isolated lignins. BioB, SeolB, OrgB; BioA and SeolA; Figure S3. DOSY spectra for BioB and BioA; Figure S4. Raw DOSY spectra for SeolA, SeolB, and OrgB; Figure S5 DOSY spectra for BioB_473_ in the aliphatic region; Figure S6. Aliphatic region for the DOSY spectra of OrgB, OrgB_463_ and OrgB_HTC2.5_; Figure S7. DOSY in the aliphatic region for SeolA, SeolA_493_ and SeolA_493-Ru_. DOSY in the aromatic region for SeolA, SeolA_523_ and SeolA_523-Ru_; Figure S8. DOSY spectra and SEC for OrgB obtained using ethanol and isopropyl alcohol; Figure S9. SEC chromatograms for the depolymerization of OrgB with NaOH in ethanol at 463 K and HTC-2.5 in ethanol at 543 K; SI. 5. HSQC and HSQC-TOCSY spectra; HSQC spectra for SeolA; Figure S11. HSQC spectra for OrgB; Figure S12. HSQC spectra for SeolB; Figure S13. HSQC spectra for acetylated BioB, BioA and HSQC-TOCSY for BioA; Figure S14. Aliphatic region in the HSQC spectra for SeolA_523_ and SeolA_523-Ru_; Figure S15. HSQC spectra for in the aliphatic region for OrgB_HTC2.5_.

## Abbreviations

BET	Brunauer-Emmett-Teller
BJH	Barrett–Joyner–Halenda
Cat.	Catalyst
D	Diffusion Coefficient
DMSO-*d_6_*	Deuterated dimethylsulfoxide
DOSY	Diffusion Ordered Spectroscopy
G	Guaiacyl units
G/S	guaiacyl to syringyl ratio
HSQC	Heteronuclear Single Quantum Coherence Spectroscopy
HTC	Hydrotalcite
ICP-OES	Inductively Coupled Plasma-Optical Emission Spectrometry
MW	Molecular weight
M_n_	Number molecular weight
M_w_	Average Molecular weight
NMR	Nuclear Magnetic Resonance
Org	Organosolv
Pd/C	Palladium supported on carbon
PEG	Poly-ethyleneglycol
PPW	*Pinus pinaster wood*
PS	Polystyrene
RCF	Reductive Catalytic Fractionation
Ru/C	Ruthenium supported on carbon
scEtOH	Supercritical ethanol
scMeOH	Supercritical methanol
S	Syringyl units
SEC	Size Exclusion Chromatography
Seos	Soda ethanosolv
S/G	Syringyl to guaiacyl ratio
wt.%	Monomer weight yield
XRD	X-Ray Diffraction

## Appendix A

Index for the discussed samples

**BioA**	Pine downstream biorefinery lignin
**BioA_473_**	**BioA** reacted with NaOH in H_2_O at 473 K
**BioB**	Poplar downstream biorefinery lignin
**BioB_473_**	**BioB** reacted with NaOH in H_2_O at 473 K
**OrgB**	Poplar Organosolv lignin obtained with isopropanol
**OrgB (EtOH)**	Poplar Organosolv lignin obtained with ethanol
**OrgB_463_**	**OrgB** reacted with NaOH in H_2_O at 463 K
**OrgB_463-EtOH_**	**OrgB** reacted with NaOH at 463 K in ethanol
**OrgB_543-EtOH_**	**OrgB** reacted with NaOH at 543 K in ethanol
**OrgB_HTCx_**	**OrgB** reacted with HTCx at 543 K in ethanol
**OrgB_523_**	**OrgB** reacted with NaOH in EtOH/H_2_O at 523 K
**OrgB_523-Ru_**	**OrgB_523_** reacted with Ru/C in EtOH at 523 K
**OrgB_Ru_**	**OrgB** reacted with Ru/C in EtOH at 523 K
**SeolA**	Pine lignin obtained by autohydrolysis and soda ethanosolv
**SeolA_493_**	**SeolA** reacted with NaOH in EtOH/H_2_O at 493 K
**SeolA_493-Ru_**	**SeolA_493_** reacted with Ru/C in EtOH at 523 K
**SeolA_523_**	**SeolA** reacted with NaOH in EtOH/H_2_O at 523 K
**SeolA_523-Ru_**	**SeolA_523_** reacted with Ru/C in EtOH at 523 K
**SeolA_Ru_**	**SeolA** reacted with Ru/C in EtOH at 523 K
**SeolB**	Poplar lignin obtained by autohydrolysis and soda ethanosolv
**SeolB_523_**	**SeolB** reacted with NaOH in EtOH/H_2_O at 523 K
**SeolB_523-Ru_**	**SeolB_523_** reacted with Ru/C in EtOH at 523 K
**SeolB_-Ru_**	**SeolB** reacted with Ru/C in EtOH at 523 K
