# Supplementary material for: Production of Aromatic Compounds by Catalytic Depolymerization of Technical and Downstream Biorefinery Lignins
Source: Biomolecules. 2020 Sep 18;10(9):1338. doi: 10.3390/biom10091338 (PMC7564559; doi:10.3390/biom10091338)
Supplement: Supplementary file 1 [file biomolecules-10-01338-s001.pdf]

# Supporting information

## Bio-based aromatic compounds production by catalytic lignin depolymerization: Assessment of technical and downstream biorefinery lignins as raw material.

**Alfonso Cornejo [1*], Fernando Bimbela [1*], Rui Moreira [2], Karina Hablich [1], Íñigo García-Yoldi [1], Maitane Maisterra [1], António Portugal [2], Luis M. Gandía [1], Víctor Martínez-Merino [1]**

[1] Institute for Advanced Materials and Mathematics (InaMat[2]) and Department of Sciences, Universidad Pública de Navarra, Pamplona, Spain

[2] CIEPQPF, FCTUC, Department of Chemical Engineering, University of Coimbra, Rua Sílvio Lima, Pólo II – Pinhal de Marrocos, 3030-790 Coimbra, Portugal

* Correspondence: alfonso.cornejo@unavarra.es, fernando.bimbela@unavarra.es; Tel.:( +34-948-166304 (A.C.); +34-948-166259 (F.B.))

Corresponding author:

alfonso.cornejo@unavarra.es, fernando.bimbela@unavarra.es

Total number of pages: 25

Total number of Figures: 15

Total number of Tables: 3

**Table of contents**

SI. 1 XRD analyses ............ 3
Figure S1. Representative XRD diffraction patterns of the fresh and calcined NiCu-HTC samples. ............ 3
SI. 2 Chemical characterization Lignin and reaction mixtures analysis............ 4
Table S1 ............ 5
SI. 3. Experimental details for NMR characterization ............ 6
Experimental details ............ 6
Integration regions used in the determination of averaged diffusion coefficients. $R^2$ correspond to the correlation between experimental and calculated values for I using the Stejskal-Tanner equation. ............ 7
Table S2. Integration regions used in the determination of averaged diffusion coefficients. $R^2$ correspond to the correlation between experimental and calculated values for I using the Stejskal-Tanner equation. ............ 8
Table S3. Integration regions used in the determination of aromatic –OH contents and G/S ratio ............ 9
SI. 4. SEC and DOSY spectra.… ............ 11
Figure S2. Normalized SEC chromatograms for isolated lignins. a) BioB, SeolB and OrgB; b) BioA and SeolA. ............ 11
Figure S3. DOSY spectra for BioB a) in the aromatic region and b) in the aliphatic region. DOSY spectra for BioA c) in the aromatic region and d) in the aliphatic region. ............ 12
Figure S4. a) Raw DOSY spectra for SeolA, SeolB and OrgB lignins. ............ 13

Figure S5 DOSY spectra for $BioB_{473}$ in the aliphatic region. ............................... 14

Figure S6. Aliphatic region for the DOSY spectra of B OrgB, $OrgB_{463}$ and $OrgB_{HTC2.5}$ .................................................................................................. 15

Figure S7. DOSY in the aliphatic region for a) SeolA, $SeolA_{493}$) and $SeolA_{493-Ru}$. b) DOSY i in the aromatic region for SeolA, $SeolA_{523}$ and $SeolA_{523-Ru}$.................... 16

Figure S8. a) DOSY spectra and b) SEC for OrgB obtained using ethanol and isopropyl alcohol. ......................................................................................... 17

Figure S9. SEC chromatograms for the depolymerization of OrgB with NaOH in ethanol at 463 K and HTC-2.5 in ethanol at 543 K................................................ 18

SI. 5. HSQC and HSQC-TOCSY spectra...................................................................... 19

Figure S10. $^{1}H$-$^{13}C$ HSQC spectra for SeolA......................................................... 19

Figure S11. $^{1}H$-$^{13}C$ HSQC spectra for OrgB ......................................................... 20

Figure S12. $^{1}H$-$^{13}C$ HSQC spectra for SeolB ........................................................ 21

Figure S13. $^{1}H$-$^{13}C$ HSQC spectra for a) acetylated BioB, b) BioA and HSQC-TOCSY for BioA........................................................................................... 22

Figure S14. Aliphatic region in the $^{1}H$-$^{13}C$ HSQC spectra for $SeolA_{523}$ and $SeolA_{523-Ru}$ ................................................................................................................ 23

Figure S15. $^{1}H$-$^{13}C$ HSQC spectra for in the aliphatic region for $OrgB_{HTC2.5}$........ 24

**SI. 1 XRD analyses**

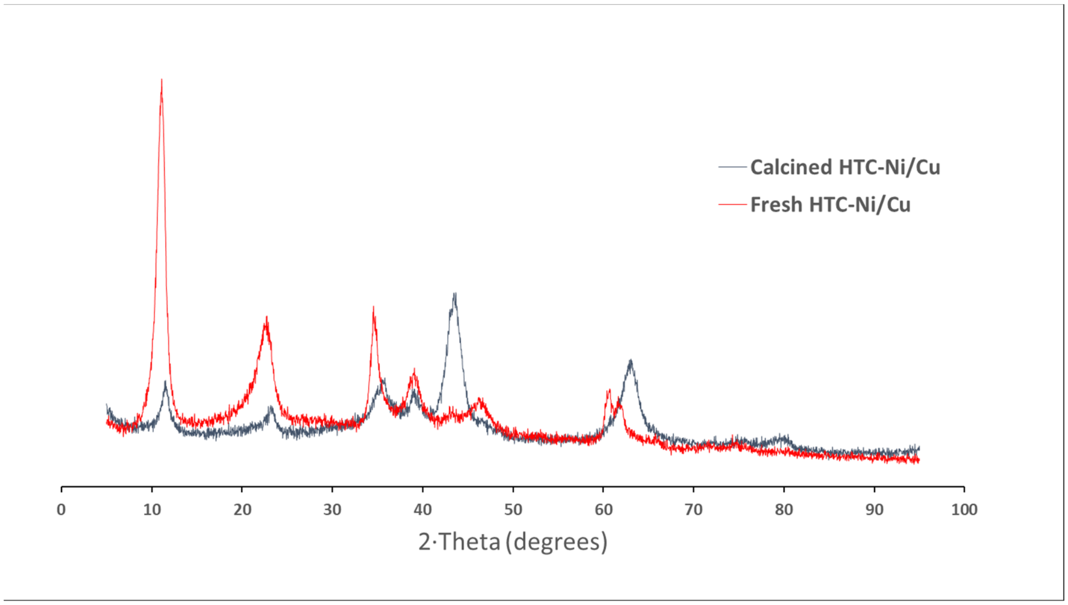


**Figure S1.** Representative XRD diffraction patterns of the fresh (red) and calcined (black) NiCu-HTC samples.

**SI. 2 Chemical characterization Lignin and reaction mixtures analysis**

Lignin content in solids was determined according to NREL/TP 510-42618. The ratio of syringyl (S) and guaiacyl (G) units, S/G ratio, was determined as described elsewhere [1,2].

The yield of monomeric phenols was quantified by GC-FID on an Agilent 6890 equipped with a capillary column HP-5MS [(5%-phenyl)-methylpolysiloxane, 60 m × 0.32 mm] with helium as carrier gas. The temperature program started at 50 °C and followed by heating to 120, 280, and 300 °C at 10 °C $min^{-1}$.·During the program, heating was held at 120 °C for 5 min, at 280 °C (8 min), and at 300 °C (2 min). Monomer yields gathers the yield of compounds showed in Table S1, whose response in FID were calibrated using bromobenzene as internal standard. Weight yield, wt.%, is referred to the weight of known monomers in the actual amount of lignin in the solid. The identity of the monomeric phenols was confirmed by GC-MS on a Shimadzu GC-2010 equipped with Shimadzu QP-2010 detector with the same analysis conditions. Although retention time was not the same in GC-FID and GC-MS the order of elution was the same.

SEC measurements were done on an Agilent 1100 equipped with RID detector. **BioA** and **BioB** were analyzed using two coupled PolarGel-M columns (300 x 7.5 mm) and PolarGel-M guard (50 x 7.5 mm) as stationary phase and 0.1 % LiBr in DMF at 40 ºC at 0.7 mL·min $-^{1}$ as eluent. **OrgB**, **SelA** and **SelB** lignins and the oily lignin products from depolymerization reactions were analyzed using Coupled HR-5 and HR-1 Styragel columns (Waters) at 30 ºC as stationary phase , THF as mobile phase at 1 mL·$min^{-1}$ flow and an injection volume of 25 µL was used. 5 mg of lignin or oily reaction mixture was dried in a vacuum desiccator at 50°C overnight and then solubilized in a 1 % v/v solution of methanol in THF before SEC analysis. Retention time was converted in molecular weight by applying a calibration curve using polystyrene. Some drops of N,N-dimethylformamide were added to **SelA** to achieve complete solubility.

**Table S1**. Aromatic monomers and retention times.

| number | Compound | Retention time (min) |
|---|---|---|
| Internal Standard | Bromobenzene | 8.67 |
| **1** | Phenol | 9.22 |
| **2** | 2-ethylphenol | 13.22 |
| **3** | o-cresol | 11.06 |
| **4-5** | m-cresol/p-cresol | 11.55/11.88 |
| **6** | Catechol | 14.89 |
| **7** | 4-methylcatechol | 17.33 |
| **8** | 4-ethylcatechol | 19.57 |
| **10** | Guaiacol | 12.26 |
| **11** | 4-methylguaiacol | 14.95 |
| **12** | 4-ethylguaiacol | 17.18 |
| **13** | 4-Propylguaiacol | 19.33 |
| **14** | Vanillin | 20.04 |
| **15** | 4-Hydroxy-3-methoxyphenylacetone | 22.40 |
| **16** | Homovanillyl alckohol | 23.17 |
| **17** | Methyl-homovanillate | 22.75 |
| **18** | Acetovanillone | 21.79 |
| **19** | Gigantol | 30.60 |
| **20** | 2,6 dimethoxyphenol (Syringol) | 18.88 |
| **21** | 4-methylsyringol | 20.18 |
| **22** | 4-ethylsyringol | - |
| **23** | 4-propylsyringol | - |
| **24** | Syringaldehyde | 24.50 |
| **25** | Acetosyringone | 25.60 |
| **26** | Homosyringaldehyde | 23.60 |

## SI. 3. Experimental details for NMR characterization

Experimental details

NMR measurements were made at 300 K on a Bruker Ascend III spectrometer equipped with a PABBO 5 probe, at 400 MHz and 101 MHz for $^{1}$H and $^{13}$C respectively, and were processed using Bruker Topspin 3.6.2 software. NMR samples were prepared at 1 % w/v in DMSO-$d_6$ and referenced using residual signal at 2.50 ppm and 39.52 ppm for $^{1}$H and $^{13}$C measurements [3].

**BioA** and **BioB** were acetylated for NMR measurements as described elsewhere [4]. $^{1}$H experiments were run using the *zg30* pulse program at 16 scans. Two-dimensional $^{1}$H-$^{13}$C correlation was carried out using HSQC and HSQC-TOCSY that run *hsqcetgpsi2* and *hsqcdietgpsisp.2* pulse program in an echo-antiecho acquisition mode with D1 = 1.48 s and D1= 2.0 s respectively. Assignment of $^{1}$H-$^{13}$C correlation signals was made according to those provided by Hu et *al.* [4] and Rencoret *et al.* [5].

DOSY was run using *stebpgp1s* pulse program in QF acquisition mode. Diffusion delay (*d20*) in *stebpgp1s* was optimized using residual DMSO signal keeping gradient pulse length (*p30*) constant at 1000 μs, resulting in 160-170 ms. Each pseudo-2D experiment consisted on a series of 16 spectra. DOSY experiments were made in DMSO-$d_6$ at 300 K and at fixed low concentrations, 1 % w/v. The accuracy of the gradient was checked by the determination of the diffusion coefficient of residual DMSO in DMSO-$d_6$. DOSY analysis were processed using TOPSPIN 3.6.2 software from Bruker. Once F2 were phased, automatic baseline correction was run using a 5$^{th}$ grade polynomial function. Using the T1/T2 relaxation module installed in the module permitted FID for the first spectrum (2 % gradient) to be extracted and to perform manual integration. The integration regions were exported to the relaxation module where decay values were fitted by area using the *vargrad* preinstalled function using a 5.35 G/mm as gradient calibration constant. Graphical processing was run using 'Dynamic Center v. 2.6.1' software from

Bruker. Unless otherwise stated, Stejskal-Tanner equation was fitted using the areas for the integration corresponding to the regions in Table S2 of the first spectrum corresponding to 2% gradient.

$^{31}P$ measures were made using a proton decoupled experiment running the *zgpg30* pulse program.

Integration regions used in the determination of averaged diffusion coefficients. $R^2$ correspond to the correlation between experimental and calculated values for I using the Stejskal-Tanner equation.

$$\ln\left(\frac{I}{I_0}\right) = -\gamma^2\delta^2 {G_z}^2 D\left[\Delta + \left(\frac{4\delta}{3} + 3\tau/2\right)\right]$$

**$I_0$**: intensity at very low gradient value

**I**: intensity at a given gradient value

**γ**: gyromagnetic ratio

**δ**: length of the bipolar gradient pulse

**Gz**: gradient strength

**Δ**: time between pulses (d20)

**τ**: gradient ringdown delay

**Table S2**. Integration regions used in the determination of averaged diffusion coefficients. $R^2$ correspond to the correlation between experimental and calculated values for I using the Stejskal-Tanner equation.

| | Aromatic | | Methoxy | | Aliphatic 1 | | Aliphatic 2 | |
|---|---|---|---|---|---|---|---|---|
| **Sample** | Chemical shift (ppm) | $R^2$ | | | Chemical shift (ppm) | $R^2$ | Chemical shift (ppm) | $R^2$ |
| BioA | 8.134 - 6.424 | 0.9910 | | | 4.714 - 3.730 | 0.9900 | 2.249 - 0.647 | 0.9870 |
| BioA$_{473}$ | 7.717 - 6.31 | 0.9990 | | | 2.411 - 1.966 | 0.9970 | 1.832 - 0.74 | 0.9990 |
| **BioB** | 7.474 - 6.357 | 0.5100 | | | 3.946 - 3.609 | 0.7960 | 2.384 - 1.104 | 0.9900 |
| BioB$_{473}$ | 7.690 - 6.842 | 0.9970 | | | 2.435 - 2.017 | 0.9970 | 1.617 - 0.946 | 0.9980 |
| **Orgb** | 7.262 - 6.465 | 0.9996 | 3.909 - 3.508 | 0.9997 | 2.391 - 1.852 | 0.9896 | 1.124 - 0.704 | |
| Blank | 7.262 - 6.465 | 0.9988 | 3.909 - 3.508 | 0.9990 | 2.391 - 1.852 | 0.9946 | 1.124 - 0.704 | 0.9985 |
| OrgB$_{HTC0}$ | 7.262 - 6.465 | 0.9998 | 3.909 - 3.509 | 0.9998 | 2.391 - 1.853 | 0.9976 | 1.124 - 0.705 | 0.9992 |
| OrgB$_{HTC1}$ | 7.262 - 6.465 | 0.9987 | 3.909 - 3.510 | 0.9987 | 2.391 - 1.854 | 0.9967 | 1.124 - 0.706 | 0.9950 |
| OrgB$_{HTC2.5}$ | 7.262 - 6.465 | 0.9994 | 3.909 - 3.511 | 0.9961 | 2.391 - 1.855 | 0.9973 | 1.124 - 0.707 | 0.9984 |
| OrgB$_{HTC4}$ | 7.262 - 6.465 | 0.9996 | 3.909 - 3.512 | 0.9999 | 2.391 - 1.856 | 0.9991 | 1.124 - 0.708 | 0.9993 |
| OrgB$_{HTC5}$ | 7.262 - 6.465 | 0.9996 | 3.909 - 3.513 | 1.0000 | 2.391 - 1.857 | 0.9981 | 1.124 - 0.709 | 0.9992 |
| OrgB$_{523}$ | 7.262 - 6.465 | 0.9991 | 3.909 - 3.514 | 0.4499 | 2.391 - 1.858 | 0.9960 | 1.124 - 0.710 | 0.9969 |
| OrgB$_{523\text{-}Ru}$ | 7.262 - 6.465 | 0.9995 | 3.909 - 3.515 | 0.9998 | 2.391 - 1.859 | 0.9989 | 1.124 - 0.711 | 0.9993 |
| **SeolA** | 7.262 - 6.465 | 0.9686 | 3.909 - 3.508 | 0.9897 | 2.391 - 1.852 | 0.9338 | 1.124 - 0.704 | 0.9623 |
| SeolA$_{493}$ | 7.262 - 6.465 | 0.9995 | 3.909 - 3.508 | 0.9985 | 2.391 - 1.852 | 0.9916 | 1.124 - 0.704 | 0.9948 |
| SeolA$_{493\text{-}Ru}$ | 7.262 - 6.465 | 0.9997 | 3.909 - 3.508 | 0.9996 | 2.391 - 1.852 | 0.9968 | 1.124 - 0.704 | 0.9981 |
| SeolA$_{523}$ | 7.262 - 6.465 | 0.9992 | 3.909 - 3.508 | 0.9996 | 2.391 - 1.852 | 0.9912 | 1.124 - 0.704 | 0.9978 |
| SeolA$_{523\text{-}Ru}$ | 7.262 - 6.465 | 0.9974 | 3.909 - 3.508 | 0.9834 | 2.391 - 1.852 | 0.9966 | 1.124 - 0.704 | 0.9948 |
| SeolA$_{Ru}$ | 7.262 - 6.465 | 0.9996 | 3.909 - 3.508 | 0.9996 | 2.391 - 1.852 | 0.9974 | 1.124 - 0.704 | 0.9980 |
| **SeolB** | 7.346 - 6.239 | 0.9965 | 3.965 - 3.562 | 0.9960 | 2.456 - 1.972 | 0.9778 | 1.832 - 0.564 | 0.9910 |
| SeolB$_{523}$ | 7.346 - 6.239 | 0.9993 | 3.965 - 3.562 | 0.9967 | 2.456 - 1.972 | 0.9883 | 1.832 - 0.564 | 0.9975 |
| SeolB$_{523\text{-}Ru}$ | 7.346 - 6.239 | 0.9995 | 3.965 - 3.562 | 0.9979 | 2.456 - 1.972 | 0.9940 | 1.832 - 0.564 | 0.9975 |
| SeolB$_{Ru}$ | 7.346 - 6.239 | 0.9979 | 3.965 - 3.562 | 0.9996 | 2.456 - 1.972 | 0.9953 | 1.832 - 0.564 | 0.9984 |

Determination of the amount of aromatic –OH contents and G/S ratio

A known amount of lignin or reaction mixture was derivatized according to the protocol described by Pu [6]. The derivatized sample was measured by $^{31}$P NMR and the *spectrum* was integrated in the 155-130 ppm region. Signals were assigned according to Table S3.

**Table S3.** Integration regions used in the determination of aromatic –OH contents and G/S ratio

| Chemical shift (ppm) | Assignment |
|---|---|
| 151.58-150.91 | Internal standard |
| 148.25-145.47 | Aliphatic –OH |
| 143.27-140.98 | Syringyl –OH |
| 139.89-136.75 | Guaiacyl-OH |
| 135.16-133.41 | Carboxylic acid -OH |

The percent of aromatic carbon atoms in the reaction mixtures were calculated from DOSY experiments using the reaction mixtures. Positive integration was carried from the aromatic and aliphatic regions that corresponded to the $^{1}$H integration corresponding to these regions.

This integration was corrected considering that, as an average, two aliphatic hydrogen atoms may correspond to only one carbon atom. In the aromatic region, one hydrogen atom corresponded to two or three carbon atoms in guaiacol and syringol respectively. A correction factor was calculated using the guaiacol/syringol (G/S) ratio estimated by $^{31}$P NMR that provided the average number of aromatic carbon atoms per aromatic hydrogen atom.

$$ArC_{factor} = \frac{(2*(G/_S)+3*1)}{(G/_S)+1} \quad \text{(SEc. 1)}$$

And the percernt of aromatic carbon atoms was calculated as follows:

$$\%ArC = \frac{(I_{Ar} * ArC_{factor})}{(I_{Ar} * ArC_{factor}) + (\sum I_{Al}/_{2})} \text{ (Sec.2)}$$

where G/S is the guaiacol/syringol ratio, $I_{Ar}$ corresponds to the positive integration of the aromatic region and $I_{Al}$ correspond to the integration of the aliphatic region, excluding methoxy groups region and solvents

## SI. 4. SEC and DOSY spectra

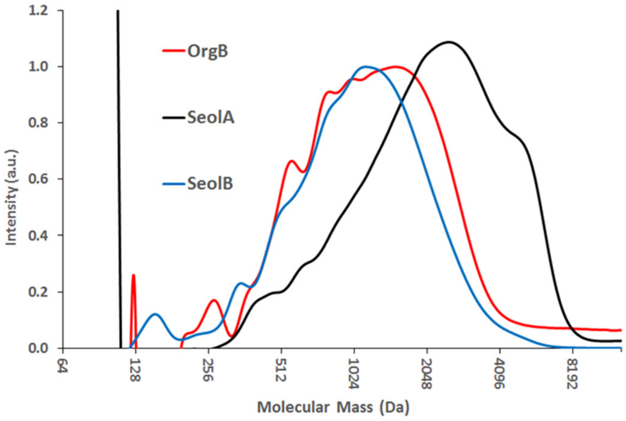


a

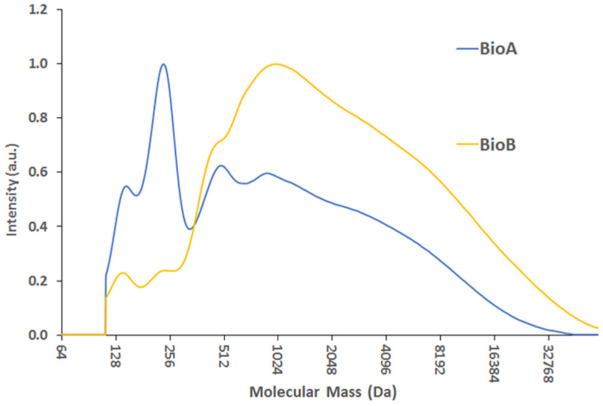


b

**Figure S2.** Normalized SEC chromatograms for isolated lignins. a) **BioB**, **SeolB** and **OrgB;** b) **BioA** and **SeolA**.

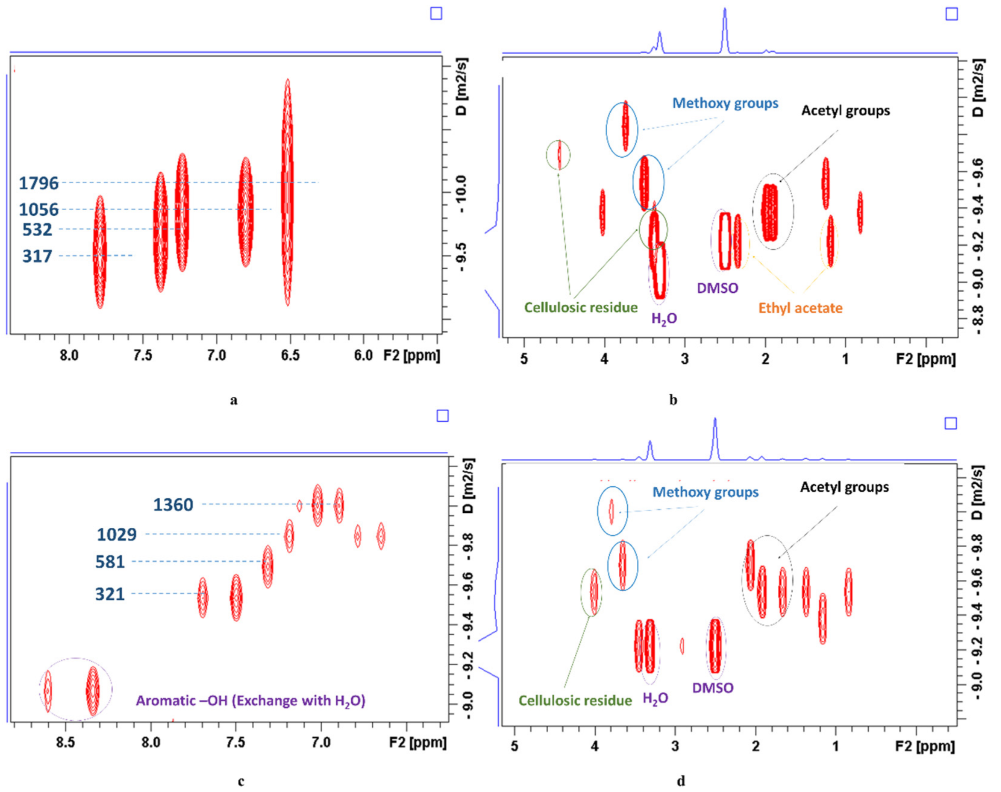


**Figure S3. DOSY** spectra for **BioB** a) in the aromatic region and b) in the aliphatic region. DOSY spectra for **BioA** c) in the aromatic region and d) in the aliphatic region. Figuress corresponded to the apparent masses in Da according to PS calibration.

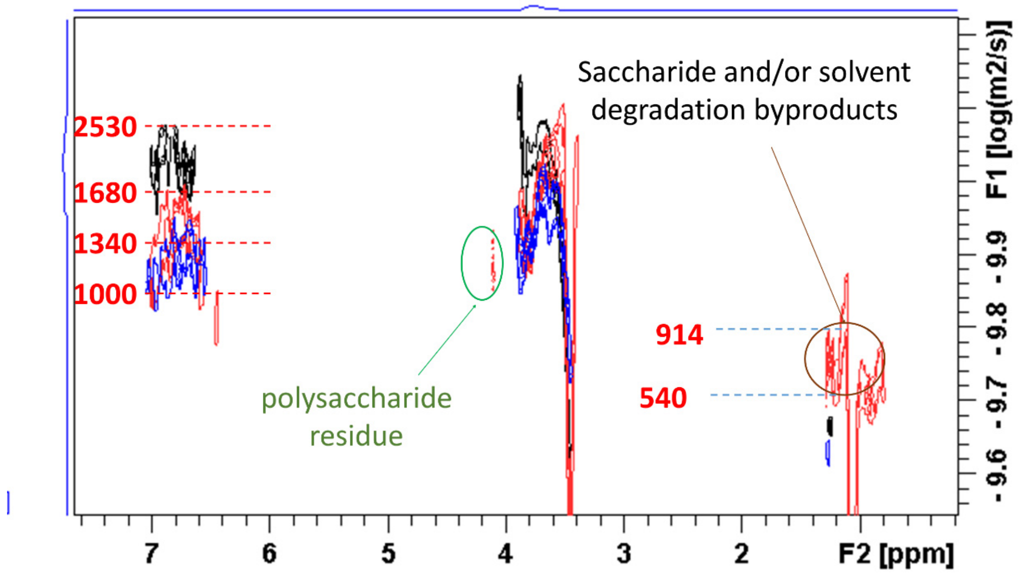


**Figure S4**. a) Raw DOSY spectra for **SeolA** (black), **SeolB** (blue) and **OrgB** (red) lignins. Red figures correspond to apparent masses according to PS calibration of log MW vs log D.

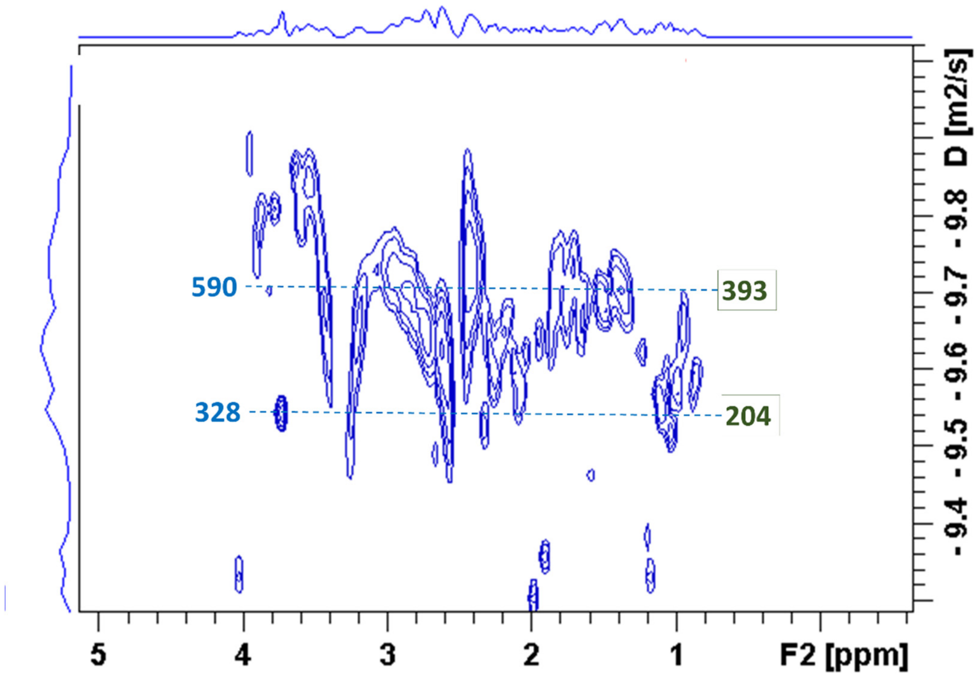


**Figure S5.** DOSY spectra for **BioB$_{473}$** in the aliphatic region. Figures in blue correspond to PS calibration Green figures correspond to PEG calibration.

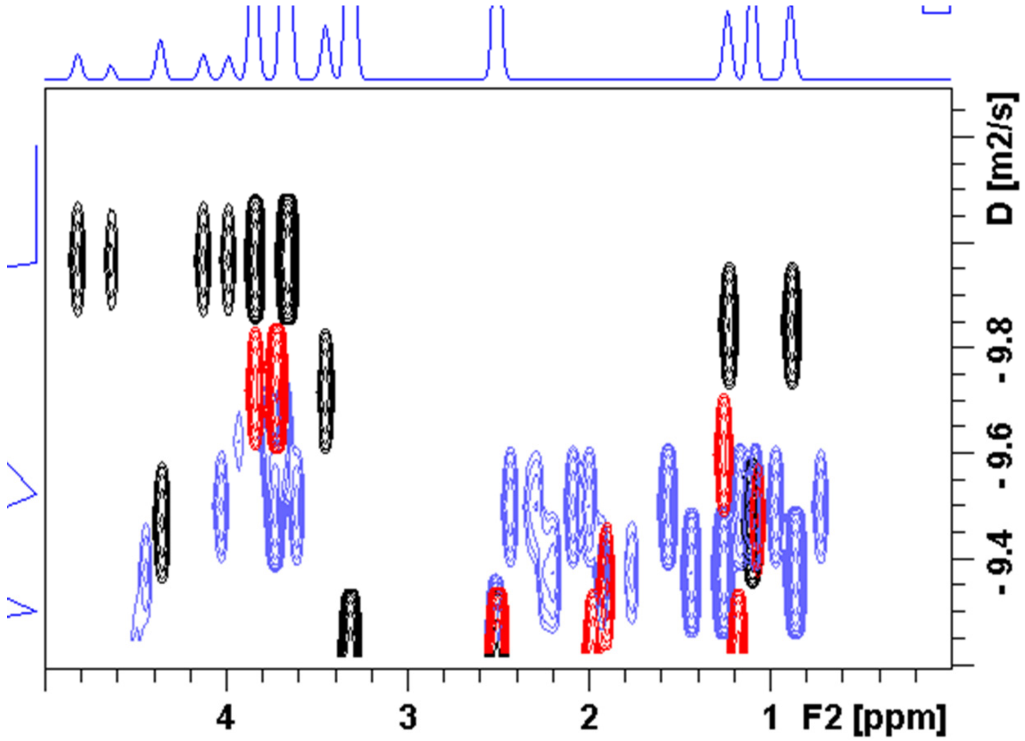


**Figure S6.** Aliphatic region for the DOSY spectra of B **OrgB** (black), **$OrgB_{463}$** (red) and **$OrgB_{HTC2.5}$** (blue).

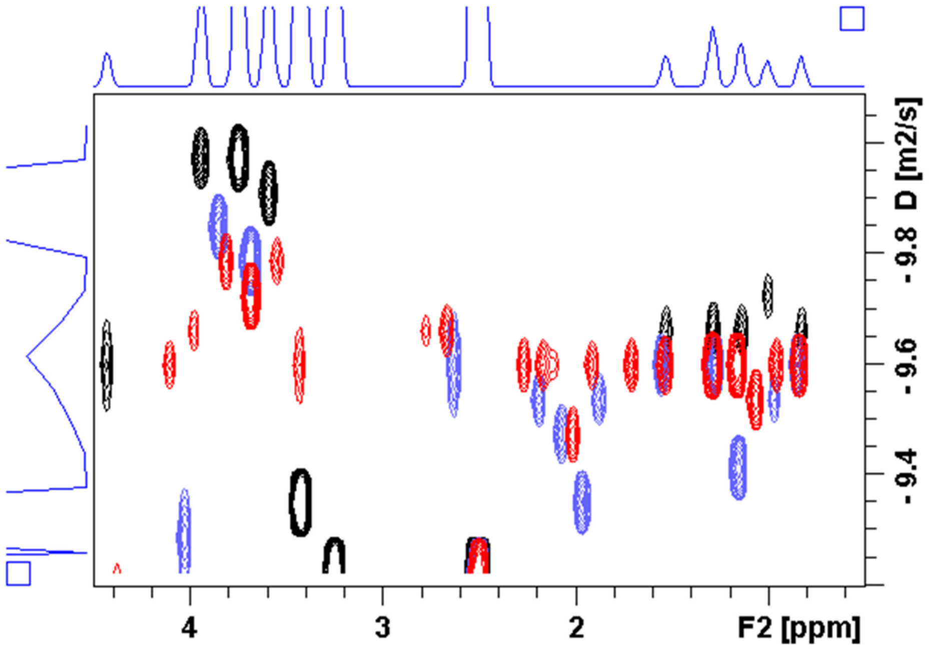


a

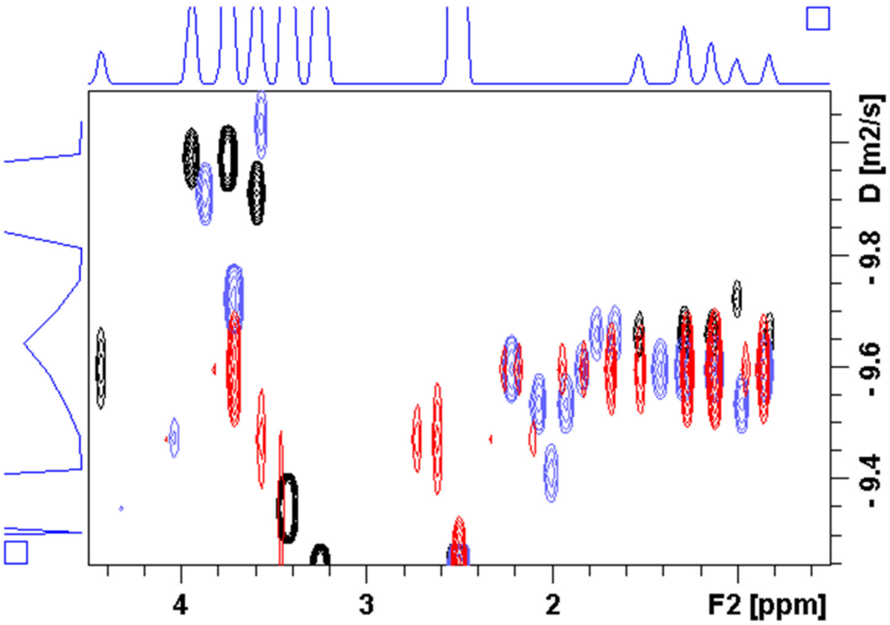


b

**Figure S7**. DOSY in the aliphatic region for a) **SeolA** (black), **SeolA$_{493}$** (blue) and **SeolA$_{493\text{-}Ru}$**. b) DOSY in the aromatic region for **SeolA** (black), **SeolA$_{523}$** (blue) and **SeolA$_{523\text{-}Ru}$** (red).

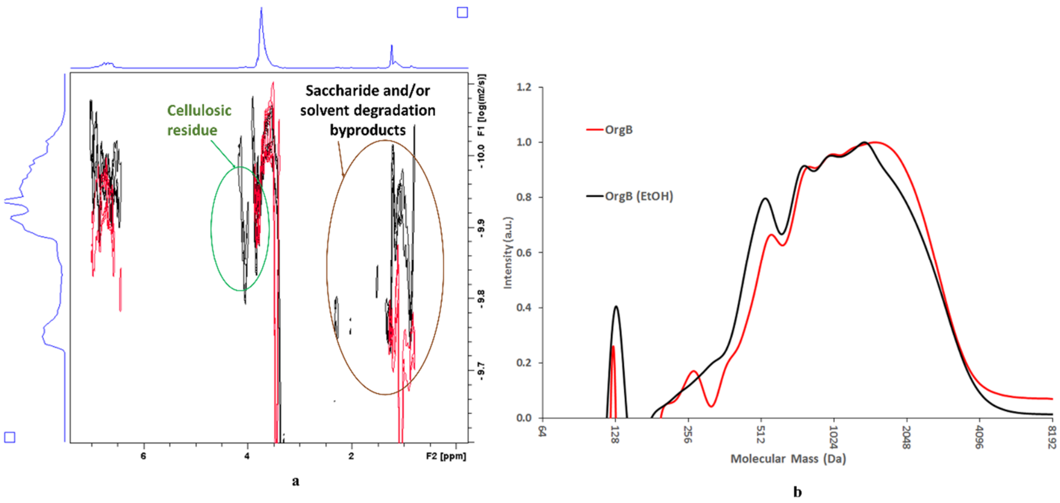


**Figure S8.** a) DOSY spectra and b) SEC for **OrgB** obtained using ethanol (black) and isopropyl alcohol (red).

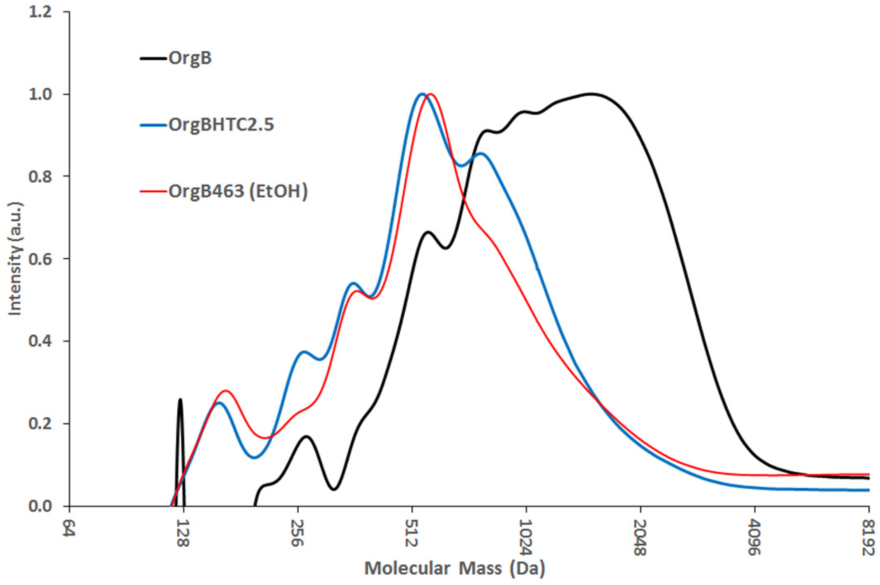


**Figure S9.** SEC chromatograms for the depolymerization of **OrgB** with NaOH in ethanol at 463 K(red) and **HTC-2.5** in ethanol at 543 K

## SI. 5. HSQC and HSQC-TOCSY spectra

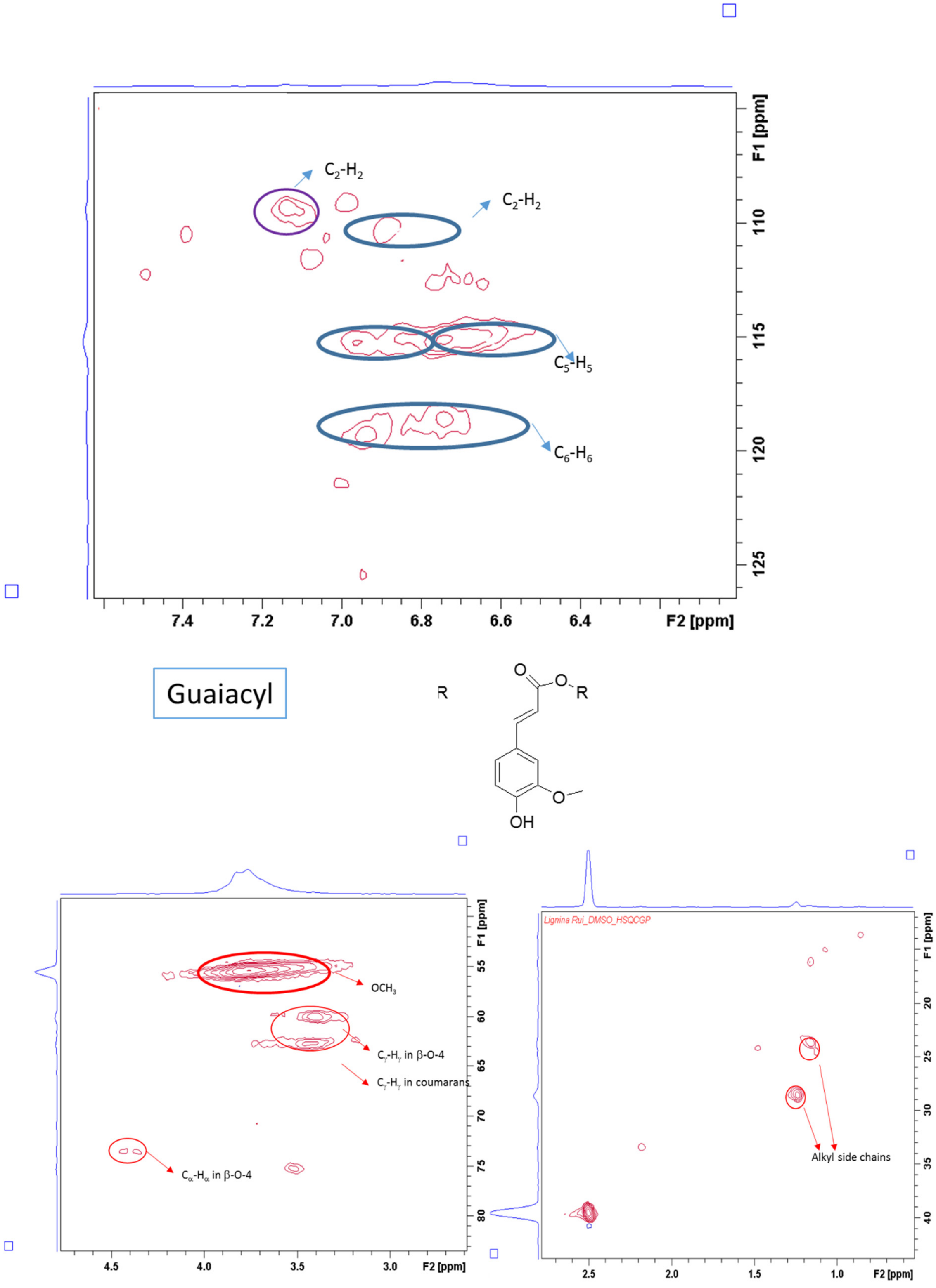


**Figure S10.** $^1$H-$^{13}$C HSQC spectra for **SeolA**

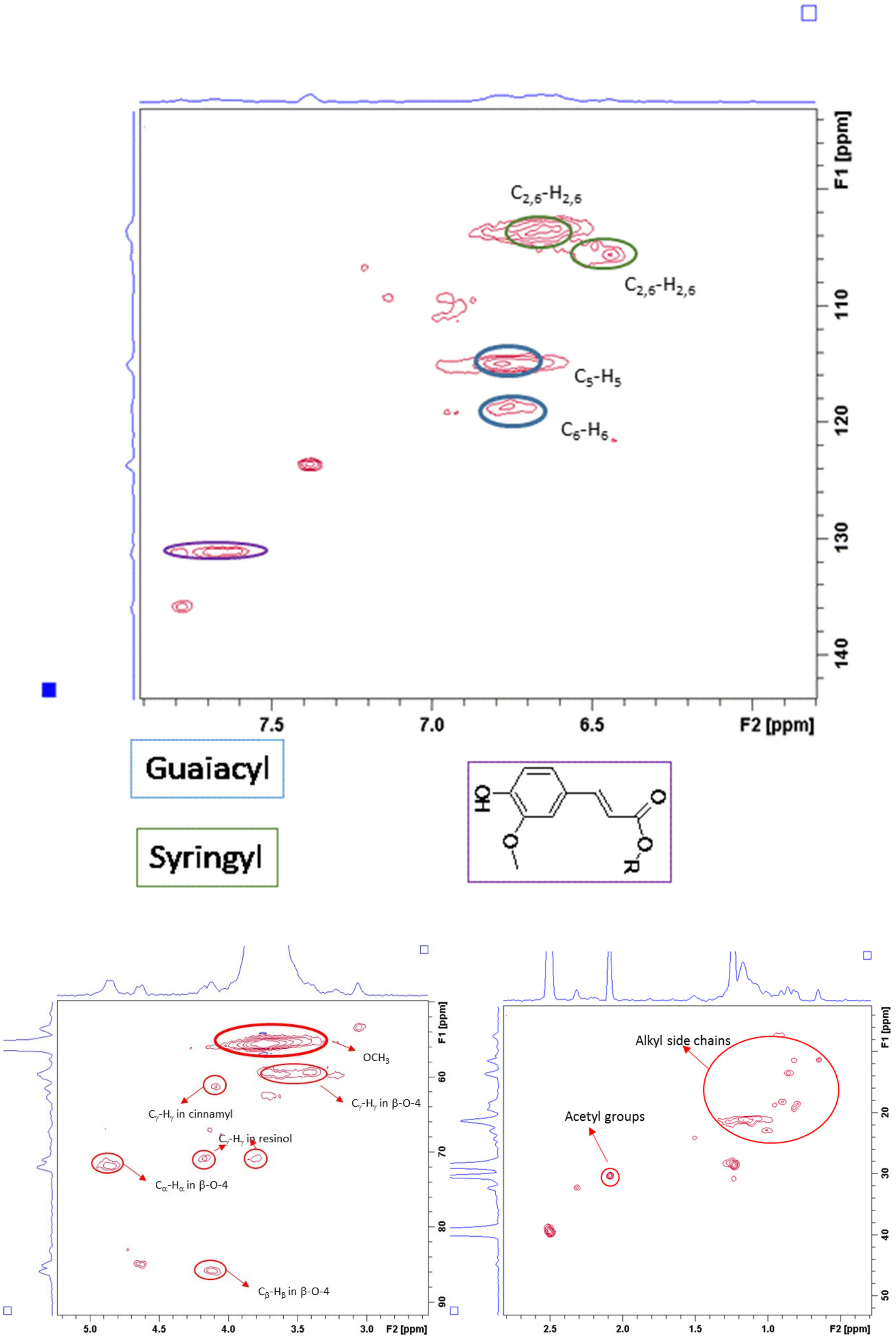


**Figure S11.** $^{1}H$-$^{13}C$ HSQC spectra for **OrgB**

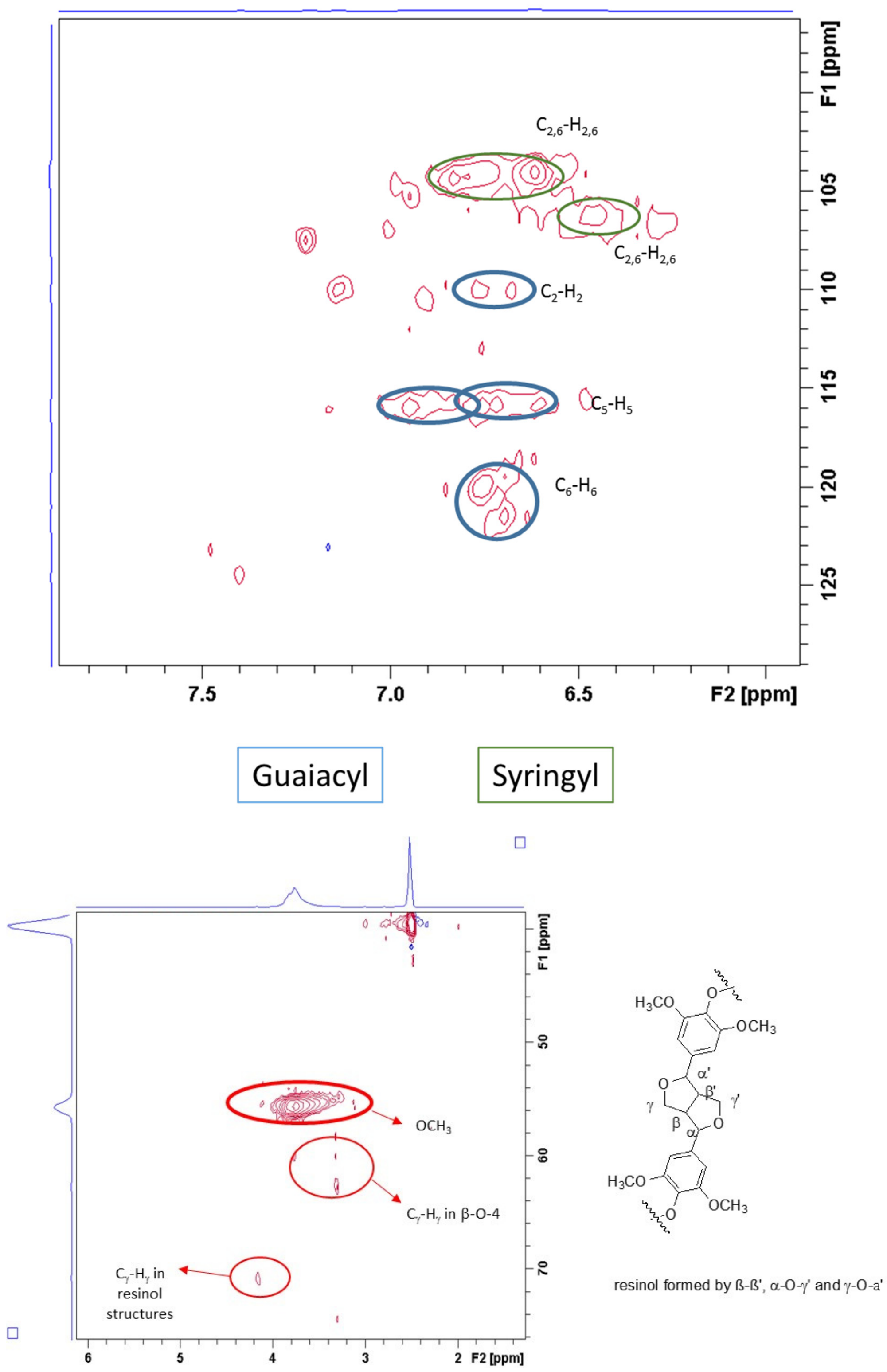


**Figure S12.** $^1$H-$^{13}$C HSQC spectra for **SeolB**

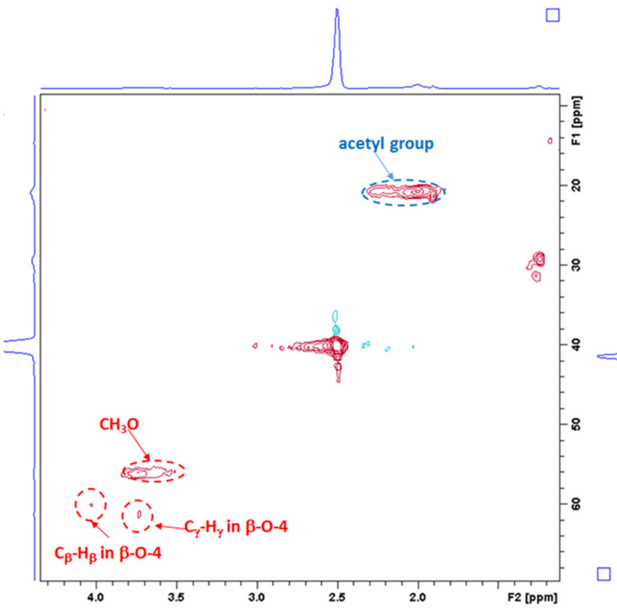


**a**

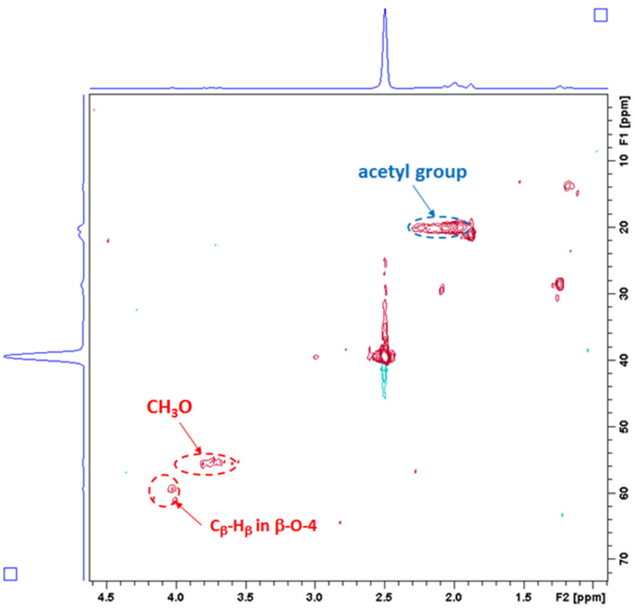


**b**

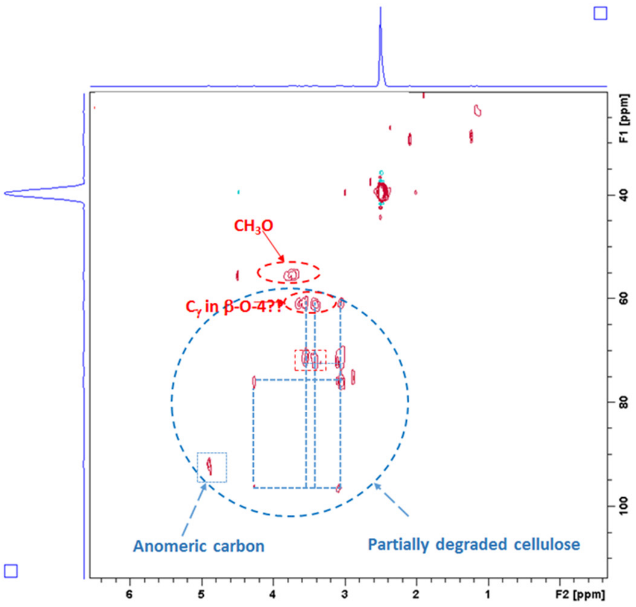


**c**

**Figure S13.** $^{1}H$-$^{13}C$ HSQC spectra for a) acetylated **BioB**, b) **BioA** and HSQC-TOCSY for **BioA**

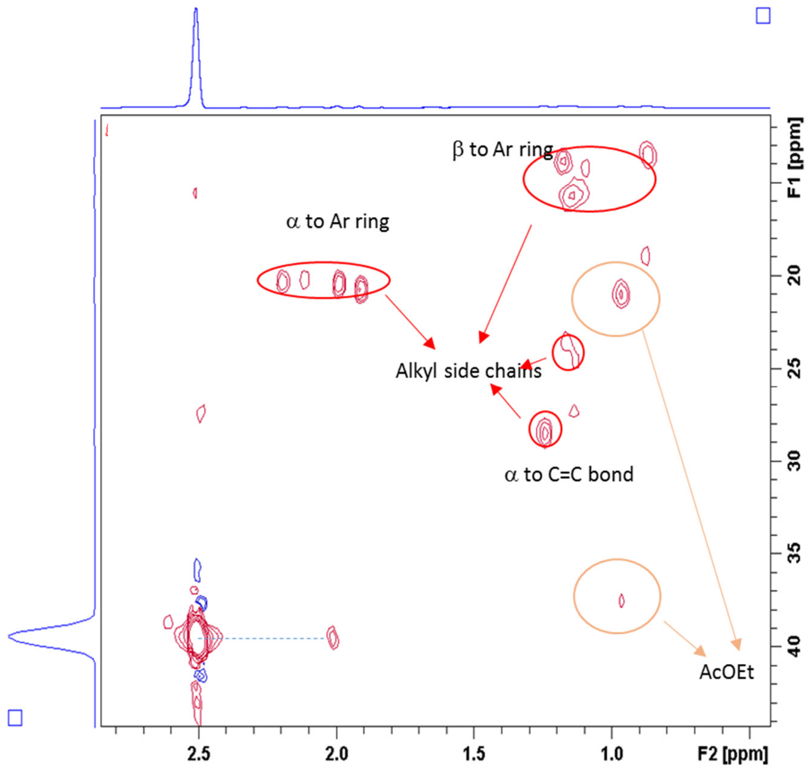


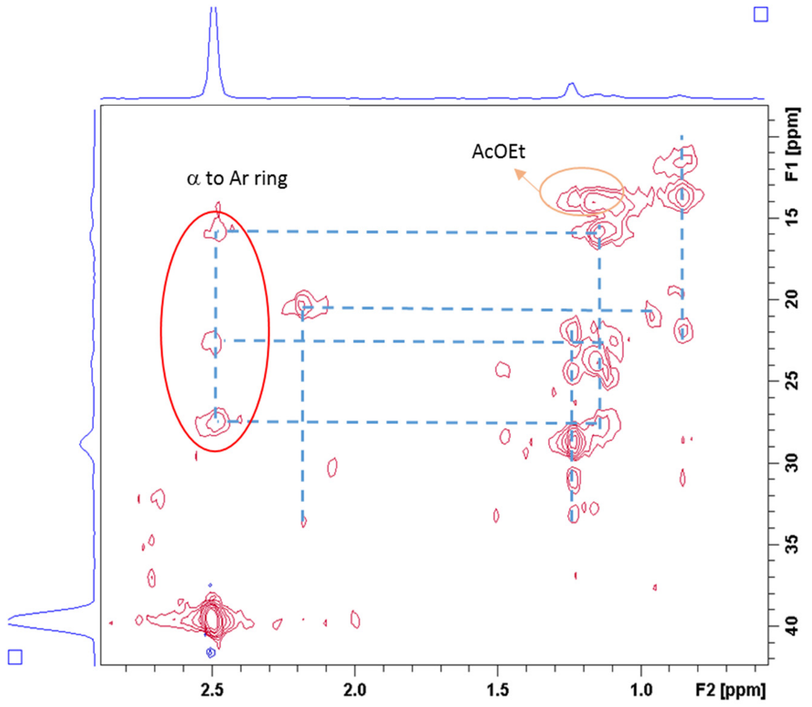


**Figure S14.** Aliphatic region in the $^{1}H$-$^{13}C$ HSQC spectra for **SeolA$_{523}$** (top) and **SeolA$_{523\text{-}Ru}$** (bottom)

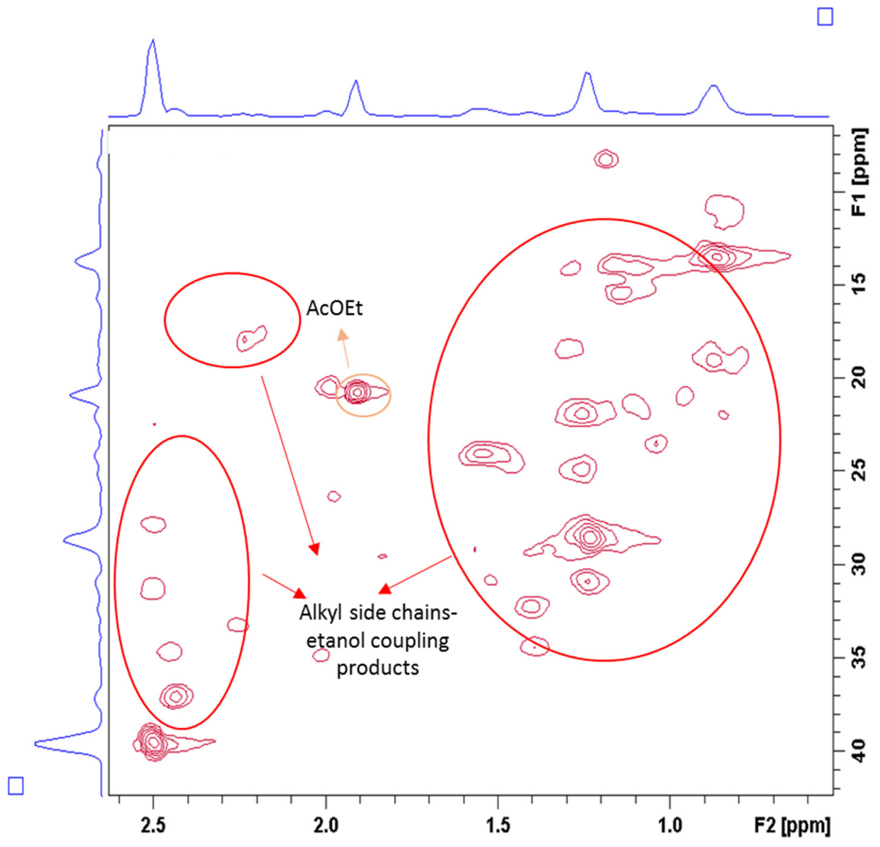


**Figure S15**. $^{1}H$-$^{13}C$ HSQC spectra for in the aliphatic region for **$OrgB_{HTC2.5}$**

References and notes


1. Ohra-Aho, T.; Gomes, F.J.B.; Colodette, J.L.; Tamminen, T. S/G ratio and lignin structure among Eucalyptus hybrids determined by Py-GC/MS and nitrobenzene oxidation. *J. Anal. Appl. Pyrolysis* **2013**, *101*, 166–171, doi:10.1016/j.jaap.2013.01.015.

2. Pinto, P.C.R.; Da Silva, E.A.B.; Rodrigues, A.E. Comparative study of solid-phase extraction and liquid-liquid extraction for the reliable quantification of high value added compounds from oxidation processes of wood-derived lignin. *Ind. Eng. Chem. Res.* **2010**, *49*, 12311–12318, doi:10.1021/ie101680s.

3. Fulmer, G.R.; Miller, A.J.M.; Sherden, N.H.; Gottlieb, H.E.; Nudelman, A.; Stoltz, B.M.; Bercaw, J.E.; Goldberg, K.I. NMR chemical shifts of trace impurities: Common laboratory solvents, organics, and gases in deuterated solvents relevant to the organometallic chemist. *Organometallics* **2010**, *29*, 2176–2179, doi:10.1021/om100106e.

4. Hu, G.; Cateto, C.; Pu, Y.; Samuel, R.; Ragauskas, A.J. Structural characterization of switchgrass lignin after ethanol organosolv pretreatment. *Energy and Fuels* **2012**, *26*, 740–745, doi:10.1021/ef201477p.

5. Rencoret, J.; Marques, G.; Gutiérrez, A.; Nieto, L.; Santos, J.I.; Jiménez-Barbero, J.; Martínez, Á.T.; Del Río, J.C. HSQC-NMR analysis of lignin in woody (Eucalyptus globulus and Picea abies) and non-woody (Agave sisalana) ball-milled plant materials at the gel state. *Holzforschung* **2009**, *63*, 691–698, doi:10.1515/HF.2009.070.

6. Pu, Y.; Cao, S.; Ragauskas, A.J. Application of quantitative 31 P NMR in biomass lignin and biofuel precursors characterization. *Energy Environ. Sci.* **2011**, *4*, 3154–3166, doi:10.1039/c1ee01201k.
